# Supplementary material for: Evolutionary Invariant of the Structure of DNA Double Helix in RNAP II Core Promoters
Source: Int J Mol Sci. 2022 Sep 17;23(18):10873. doi: 10.3390/ijms231810873 (PMC9504043; doi:10.3390/ijms231810873)
Supplement: Supplementary file 1 [file ijms-23-10873-s001.zip › s1.pdf]

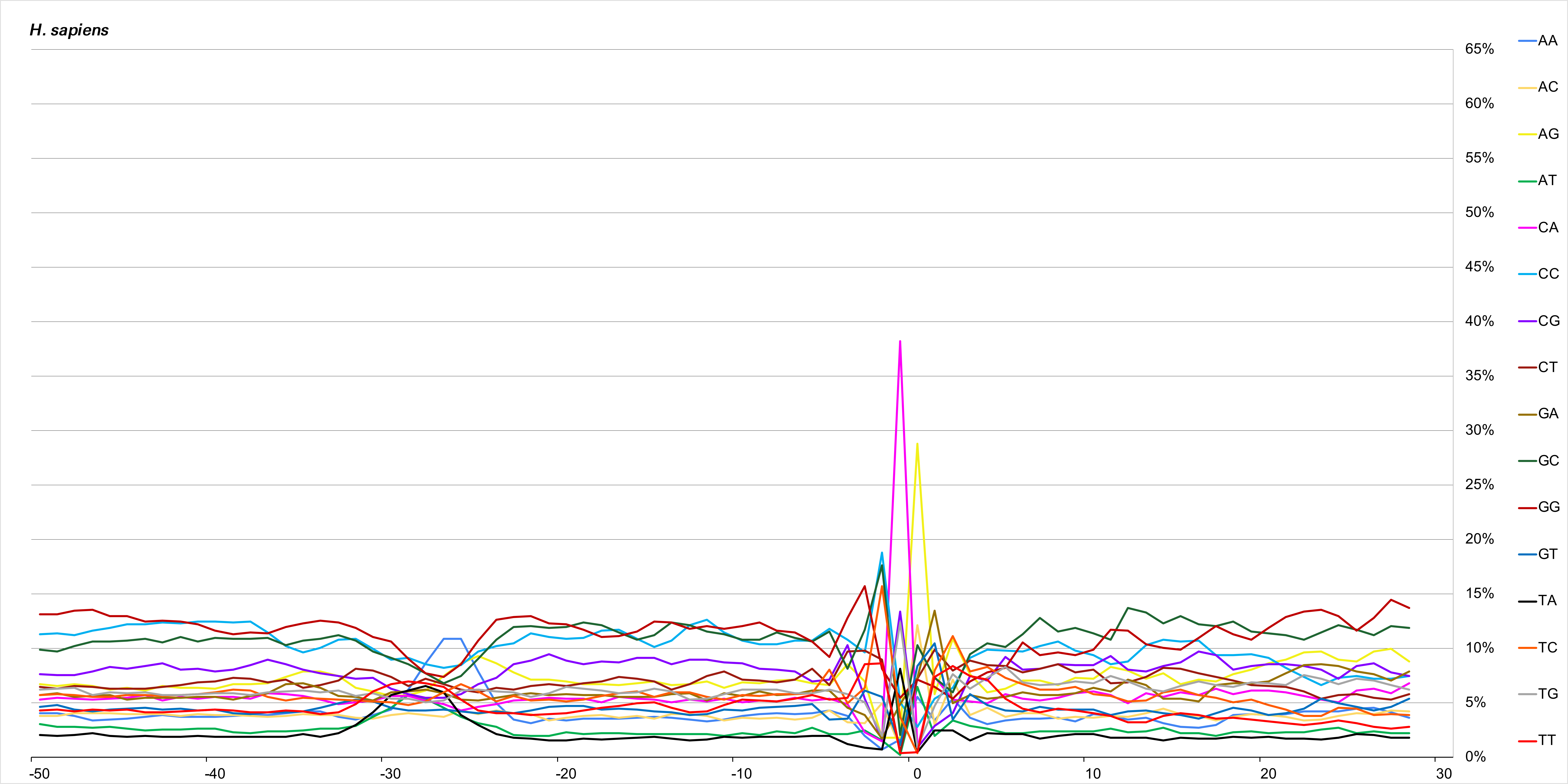

H. sapiens
65%
60%
55%
50%
45%
40%
35%
30%
25%
20%
15%
10%
5%
0%
-50
-40
-30
-20
-10
0
10
20
30
AA
AC
AG
AT
CA
CC
CG
CT
GA
GC
GG
GT
TA
TC
TG
TT

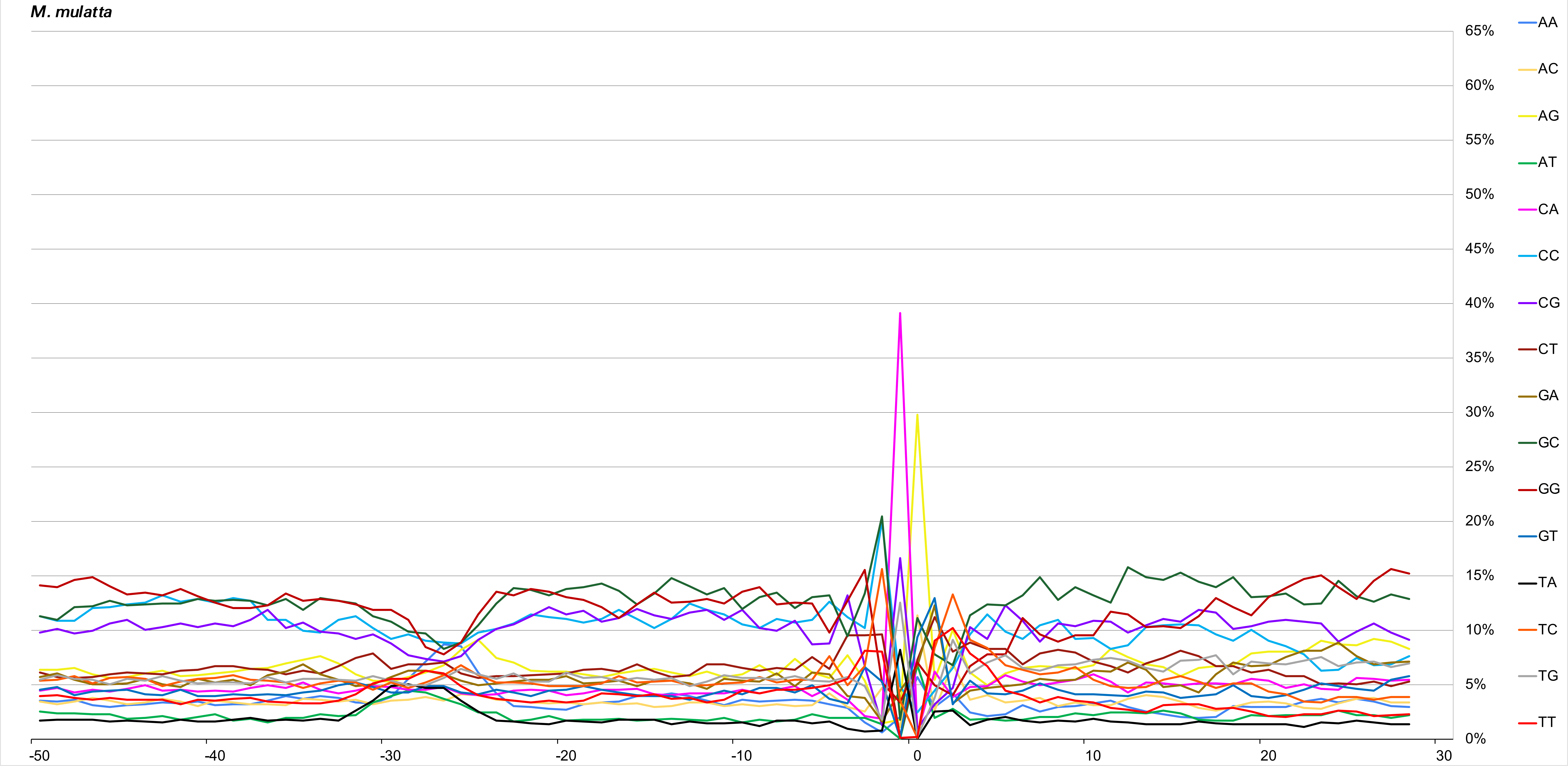

M. mulatta
65%
60%
55%
50%
45%
40%
35%
30%
25%
20%
15%
10%
5%
0%
-50
-40
-30
-20
-10
0
10
20
30
AA
AC
AG
AT
CA
CC
CG
CT
GA
GC
GG
GT
TA
TC
TG
TT

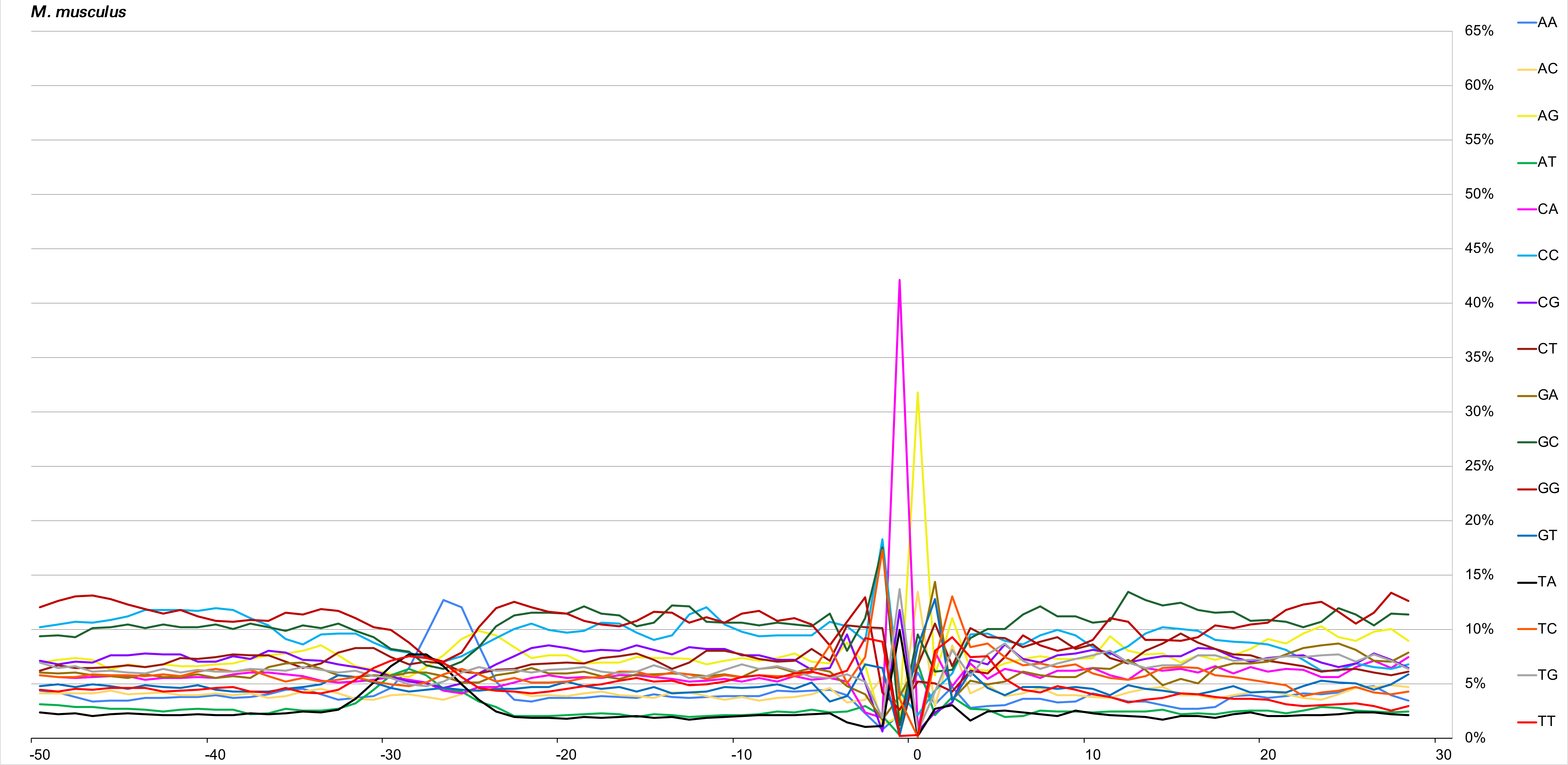

M. musculus
65%
60%
55%
50%
45%
40%
35%
30%
25%
20%
15%
10%
5%
0%
-50
-40
-30
-20
-10
0
10
20
30
AA
AC
AG
AT
CA
CC
CG
CT
GA
GC
GG
GT
TA
TC
TG
TT

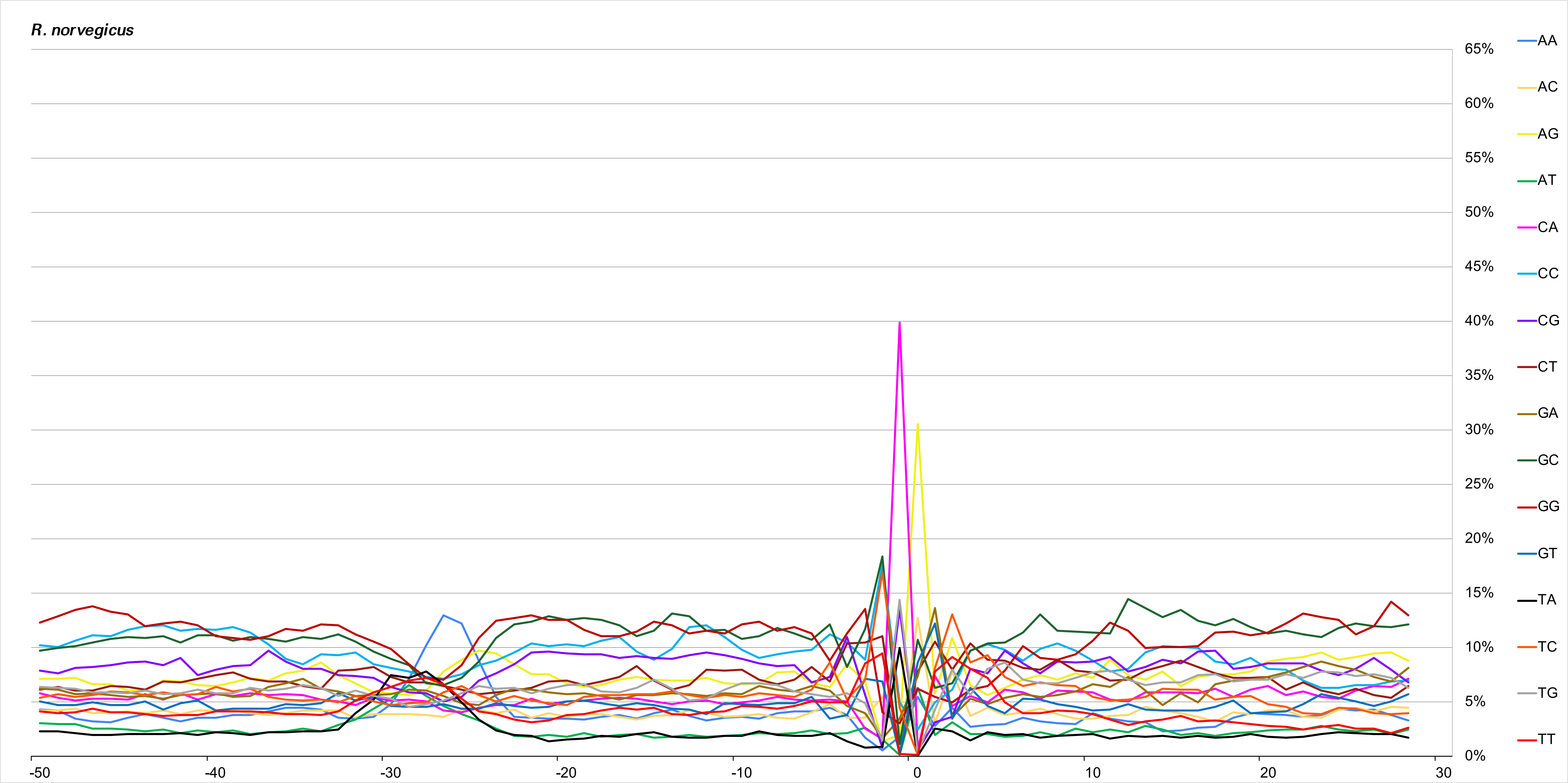

R. norvegicus
65%
60%
55%
50%
45%
40%
35%
30%
25%
20%
15%
10%
5%
0%
-50
-40
-30
-20
-10
0
10
20
30
AA
AC
AG
AT
CA
CC
CG
CT
GA
GC
GG
GT
TA
TC
TG
TT

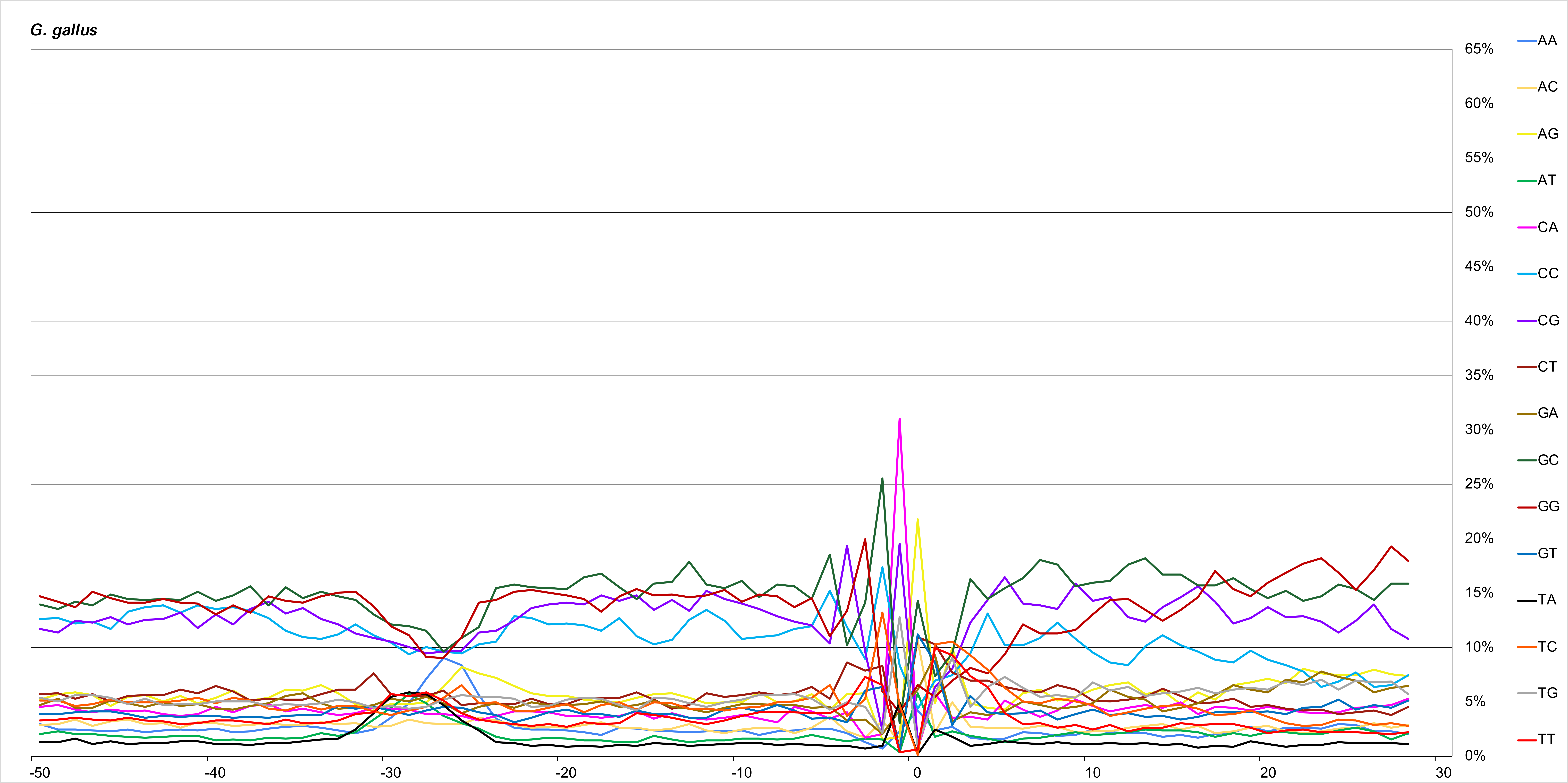
G. gallus
65%
60%
55%
50%
45%
40%
35%
30%
25%
20%
15%
10%
5%
0%
-50
-40
-30
-20
-10
0
10
20
30
AA
AC
AG
AT
CA
CC
CG
CT
GA
GC
GG
GT
TA
TC
TG
TT

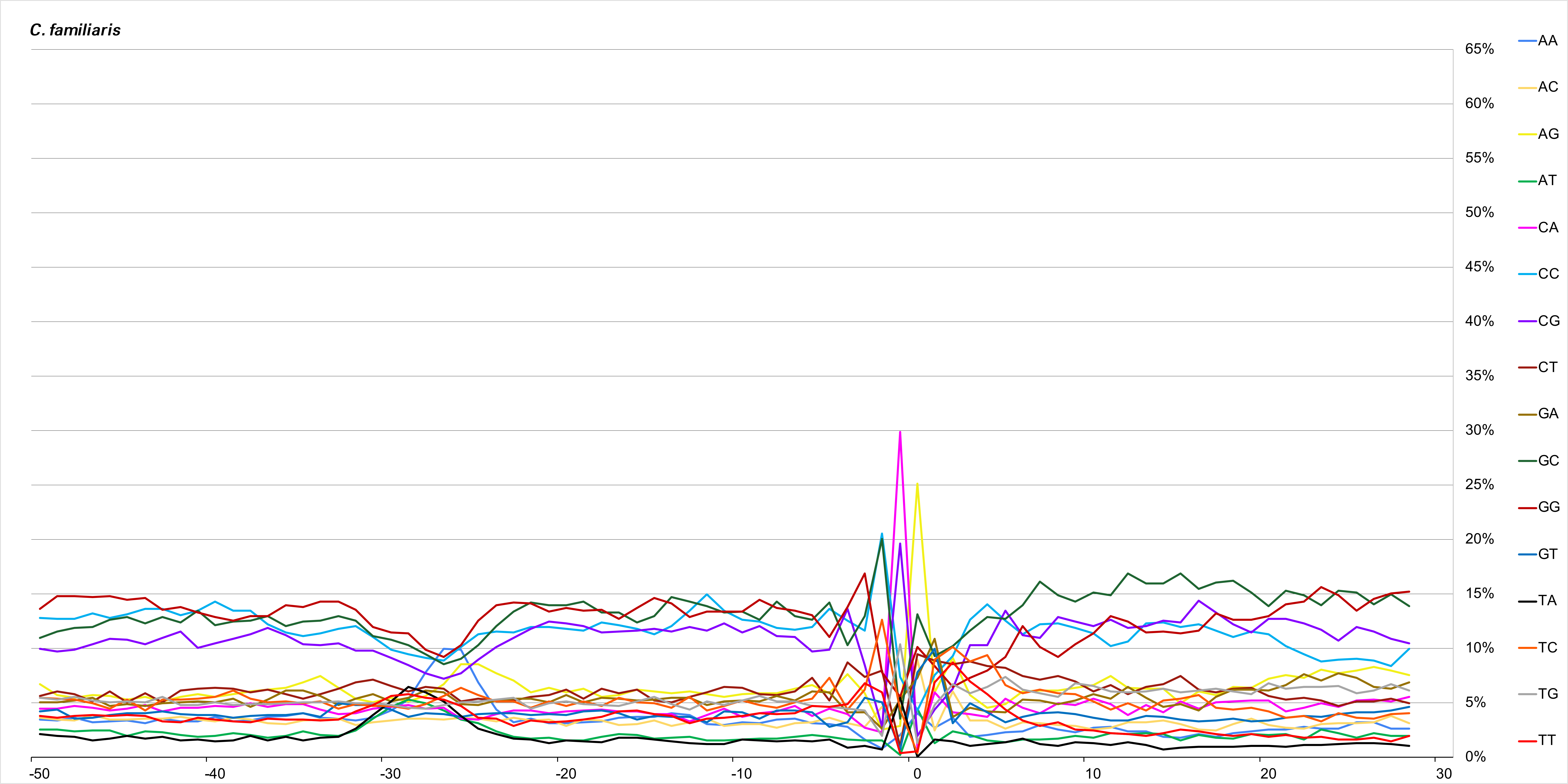
C. familiaris
65%
60%
55%
50%
45%
40%
35%
30%
25%
20%
15%
10%
5%
0%
-50
-40
-30
-20
-10
0
10
20
30
AA
AC
AG
AT
CA
CC
CG
CT
GA
GC
GG
GT
TA
TC
TG
TT

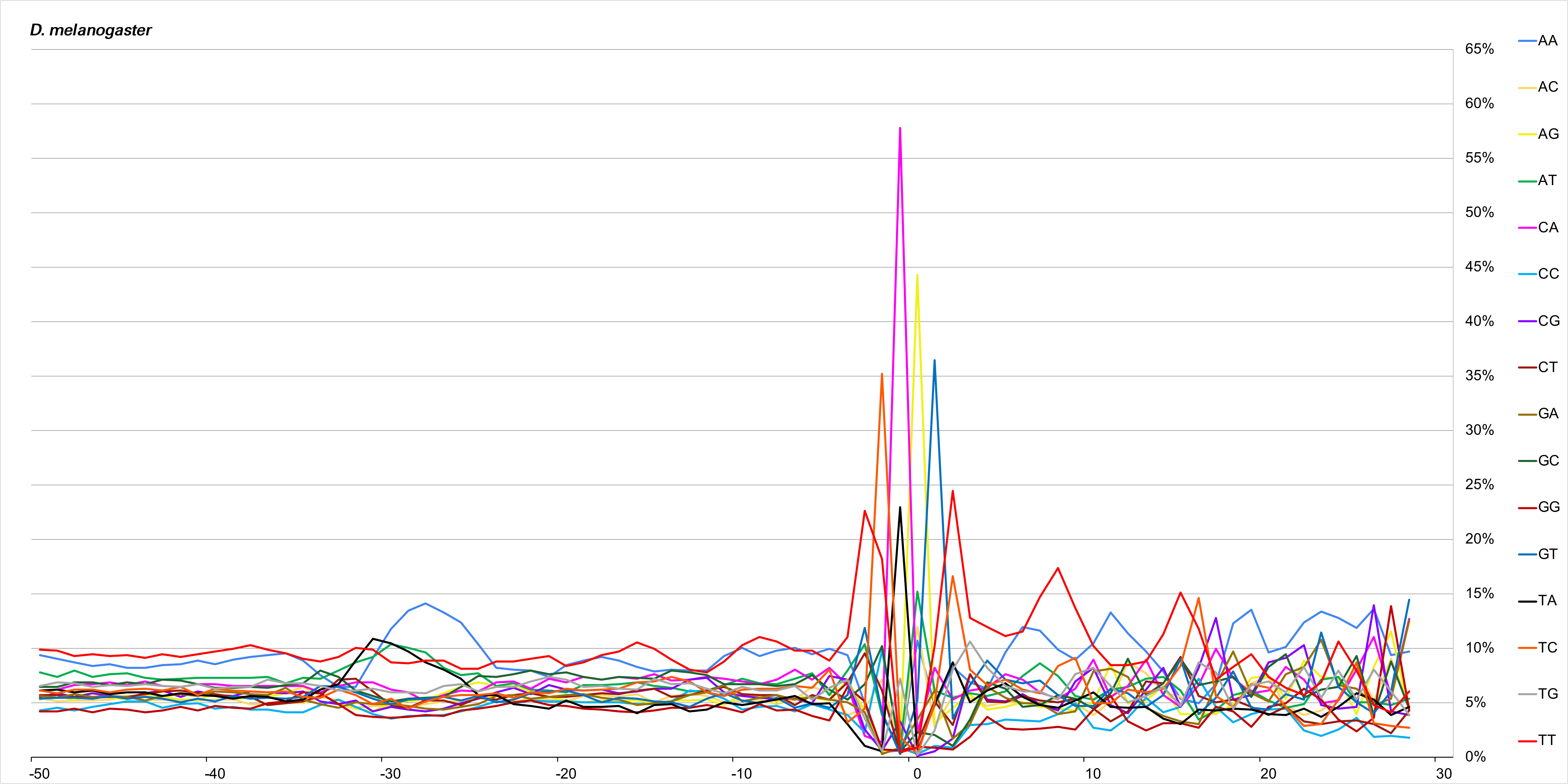

D. melanogaster
65%
60%
55%
50%
45%
40%
35%
30%
25%
20%
15%
10%
5%
0%
-50
-40
-30
-20
-10
0
10
20
30
AA
AC
AG
AT
CA
CC
CG
CT
GA
GC
GG
GT
TA
TC
TG
TT

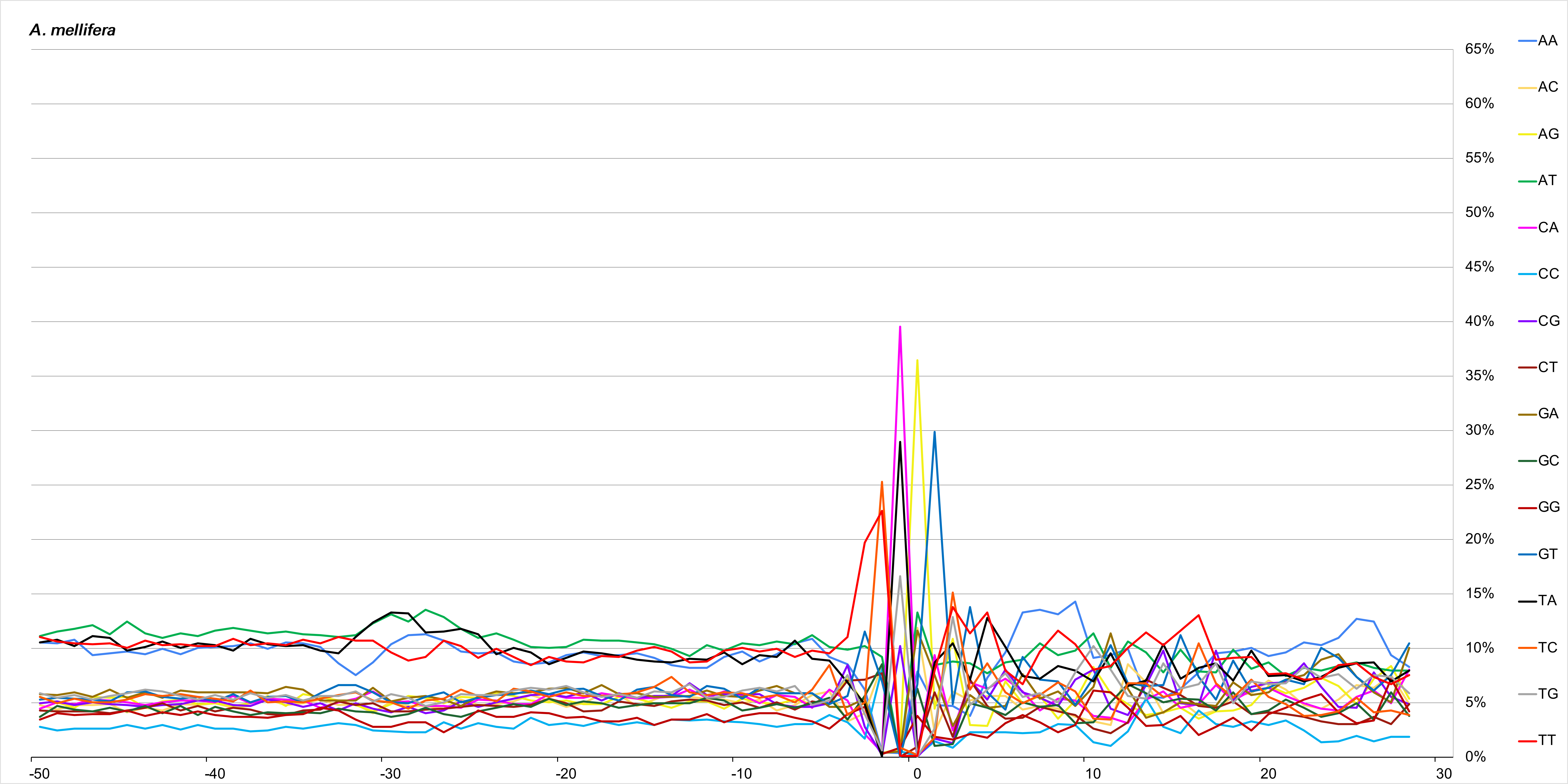

A. mellifera
65%
60%
55%
50%
45%
40%
35%
30%
25%
20%
15%
10%
5%
0%
-50
-40
-30
-20
-10
0
10
20
30
AA
AC
AG
AT
CA
CC
CG
CT
GA
GC
GG
GT
TA
TC
TG
TT

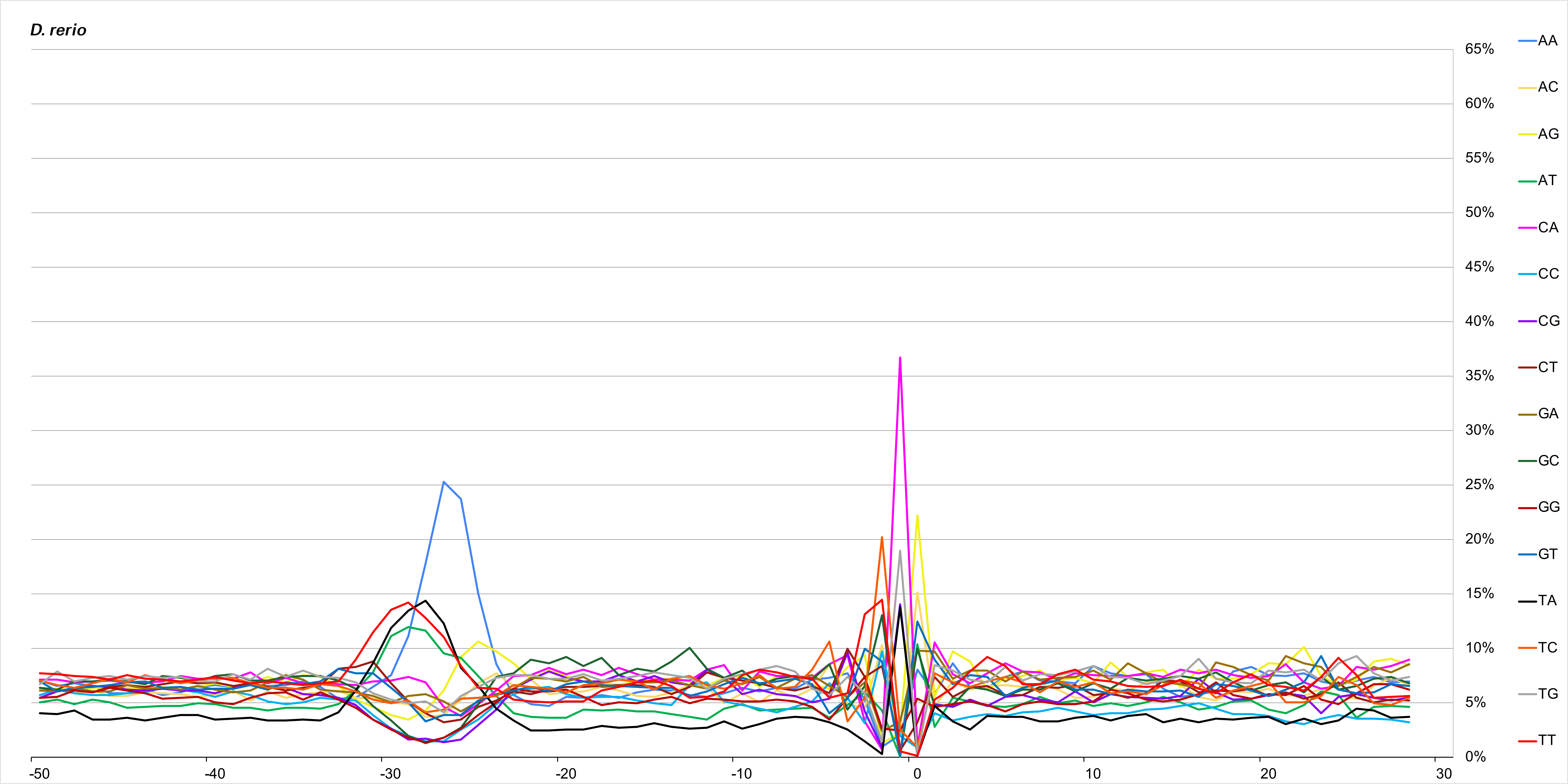

D. rerio
65%
60%
55%
50%
45%
40%
35%
30%
25%
20%
15%
10%
5%
0%
-50
-40
-30
-20
-10
0
10
20
30
AA
AC
AG
AT
CA
CC
CG
CT
GA
GC
GG
GT
TA
TC
TG
TT

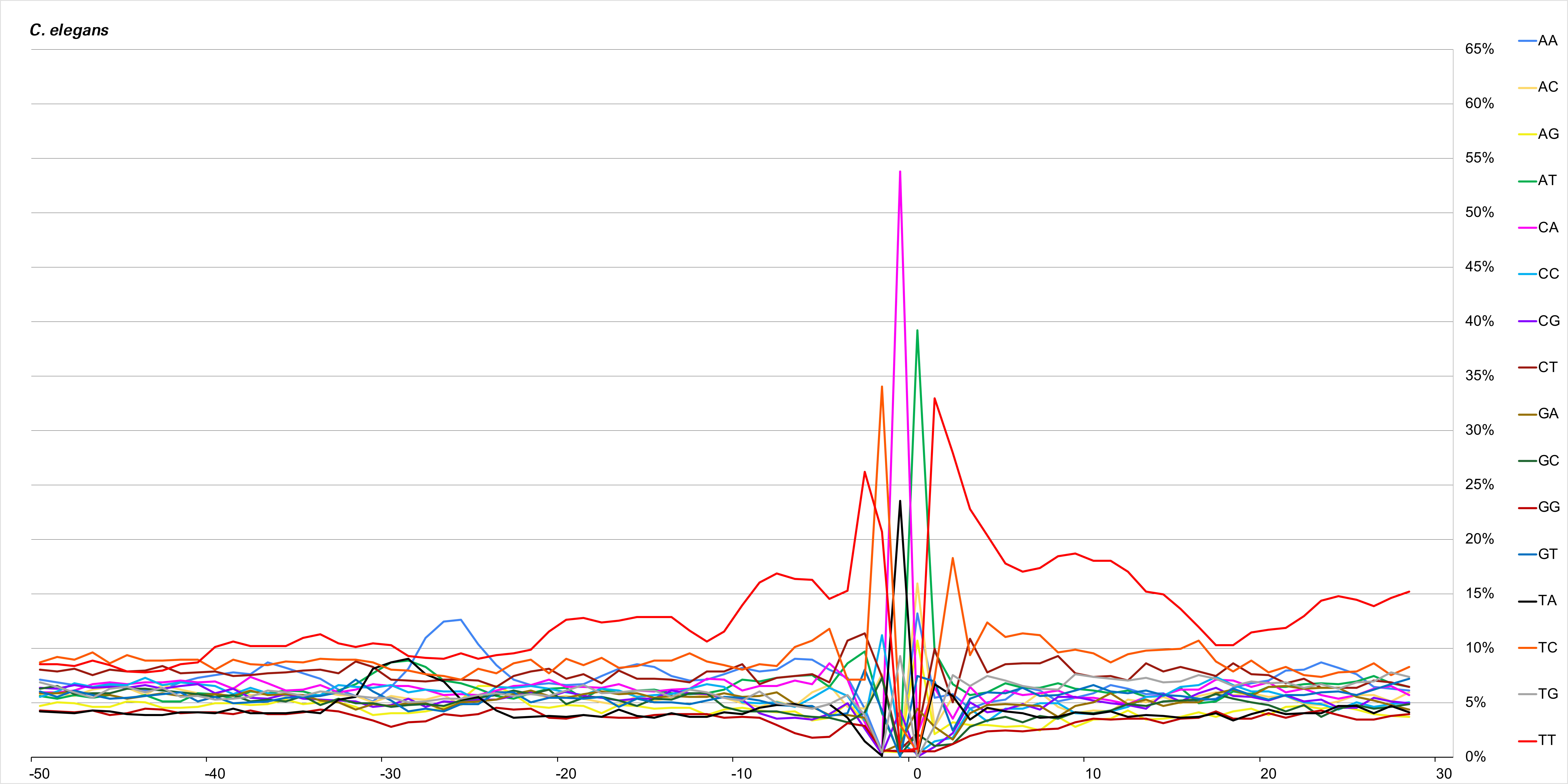

C. elegans
65%
60%
55%
50%
45%
40%
35%
30%
25%
20%
15%
10%
5%
0%
-50
-40
-30
-20
-10
0
10
20
30
AA
AC
AG
AT
CA
CC
CG
CT
GA
GC
GG
GT
TA
TC
TG
TT

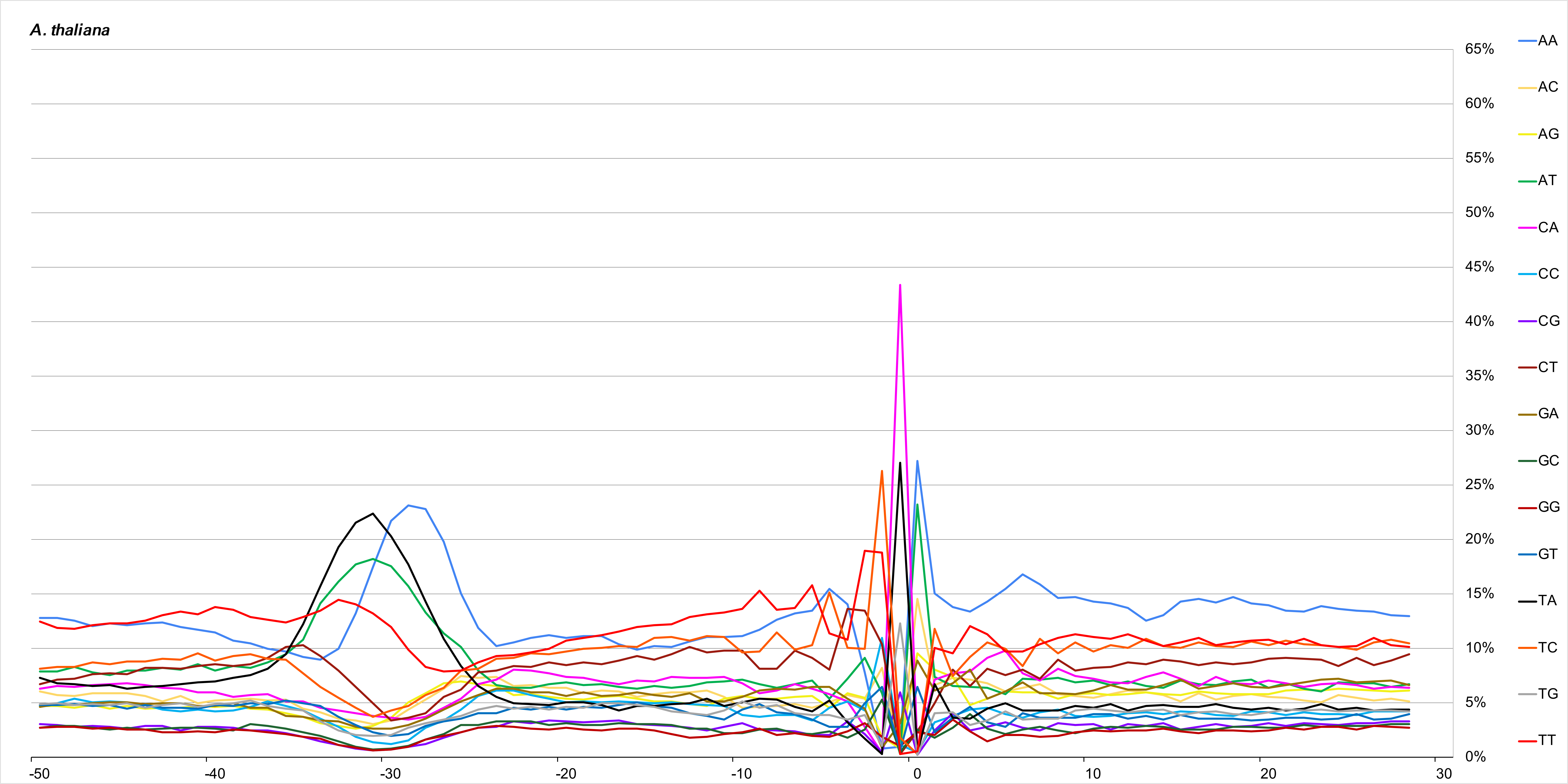

A. thaliana
65%
60%
55%
50%
45%
40%
35%
30%
25%
20%
15%
10%
5%
0%
-50
-40
-30
-20
-10
0
10
20
30
AA
AC
AG
AT
CA
CC
CG
CT
GA
GC
GG
GT
TA
TC
TG
TT

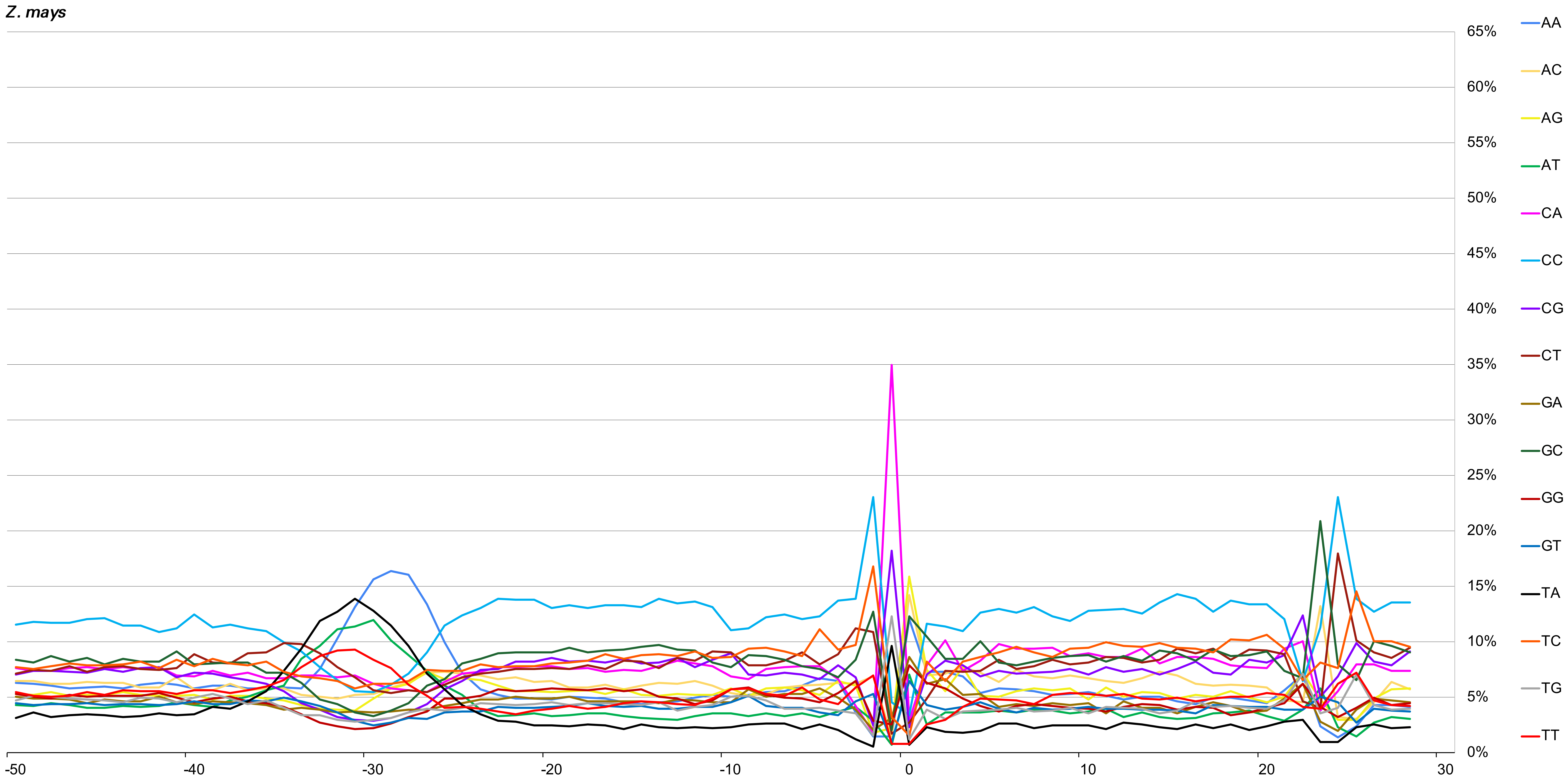

Z. mays
65%
60%
55%
50%
45%
40%
35%
30%
25%
20%
15%
10%
5%
0%
-50
-40
-30
-20
-10
0
10
20
30
AA
AC
AG
AT
CA
CC
CG
CT
GA
GC
GG
GT
TA
TC
TG
TT

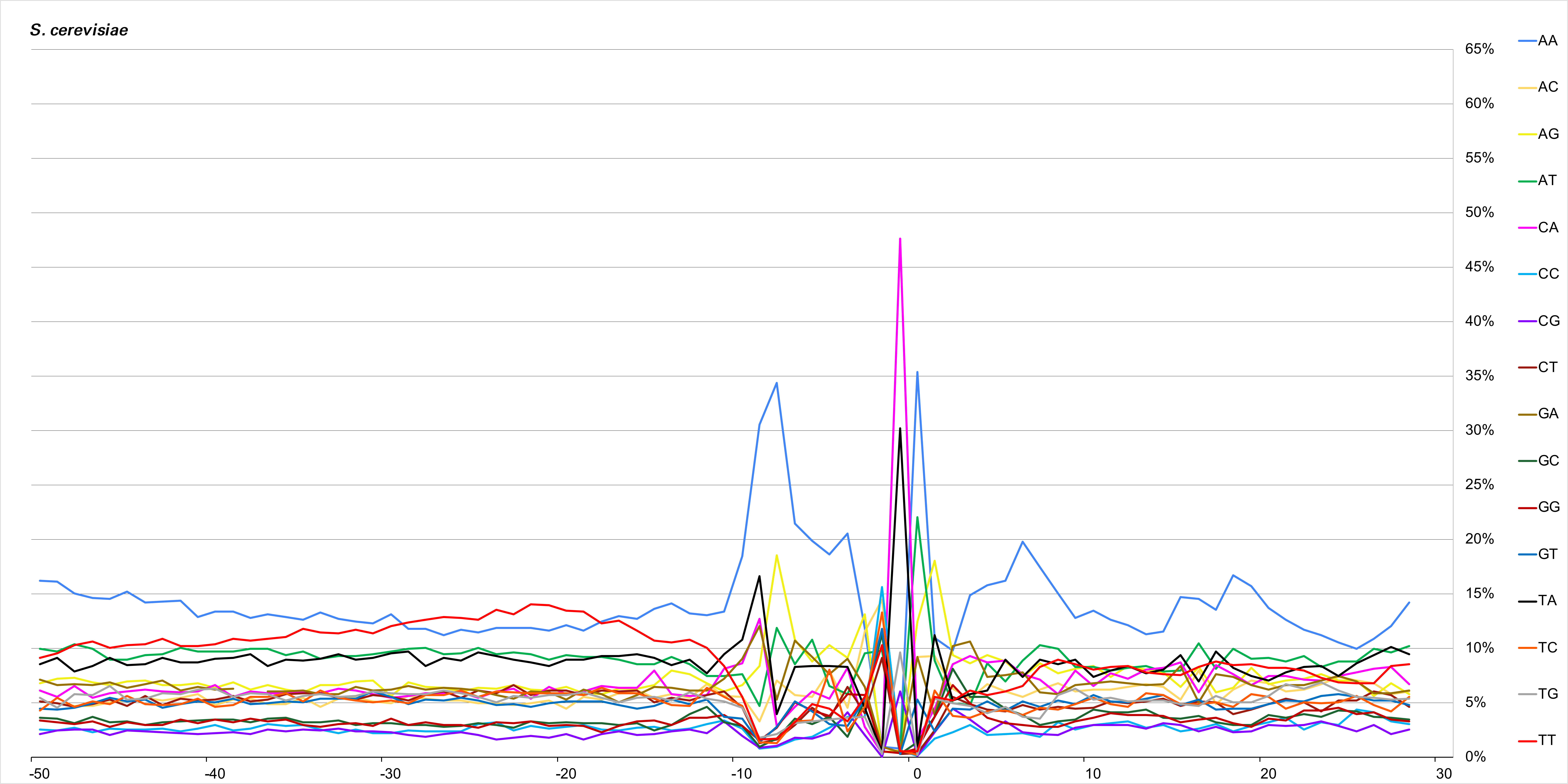

S. cerevisiae
65%
60%
55%
50%
45%
40%
35%
30%
25%
20%
15%
10%
5%
0%
-50
-40
-30
-20
-10
0
10
20
30
AA
AC
AG
AT
CA
CC
CG
CT
GA
GC
GG
GT
TA
TC
TG
TT

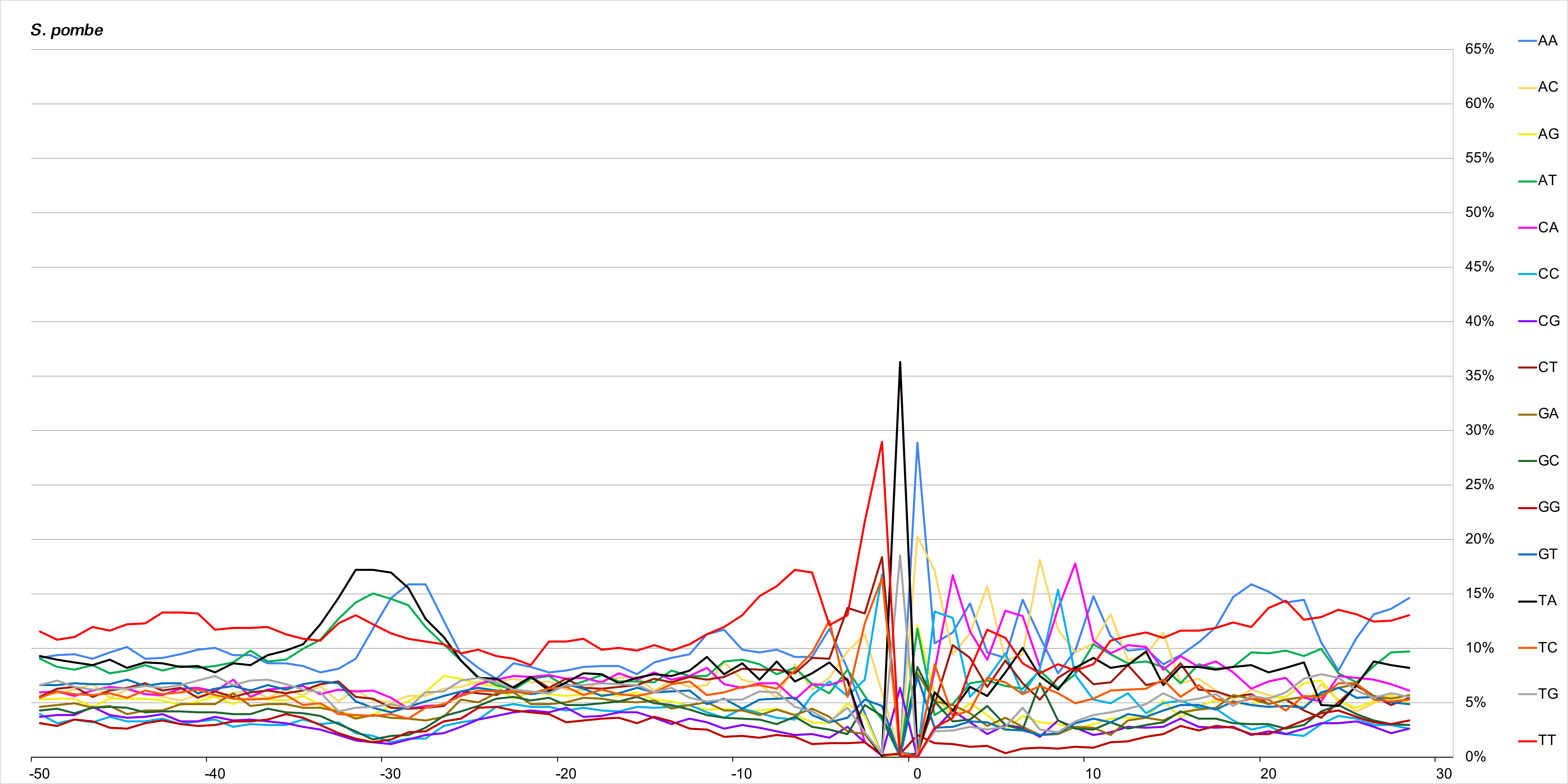

S. pombe
65%
60%
55%
50%
45%
40%
35%
30%
25%
20%
15%
10%
5%
0%
-50
-40
-30
-20
-10
0
10
20
30
AA
AC
AG
AT
CA
CC
CG
CT
GA
GC
GG
GT
TA
TC
TG
TT

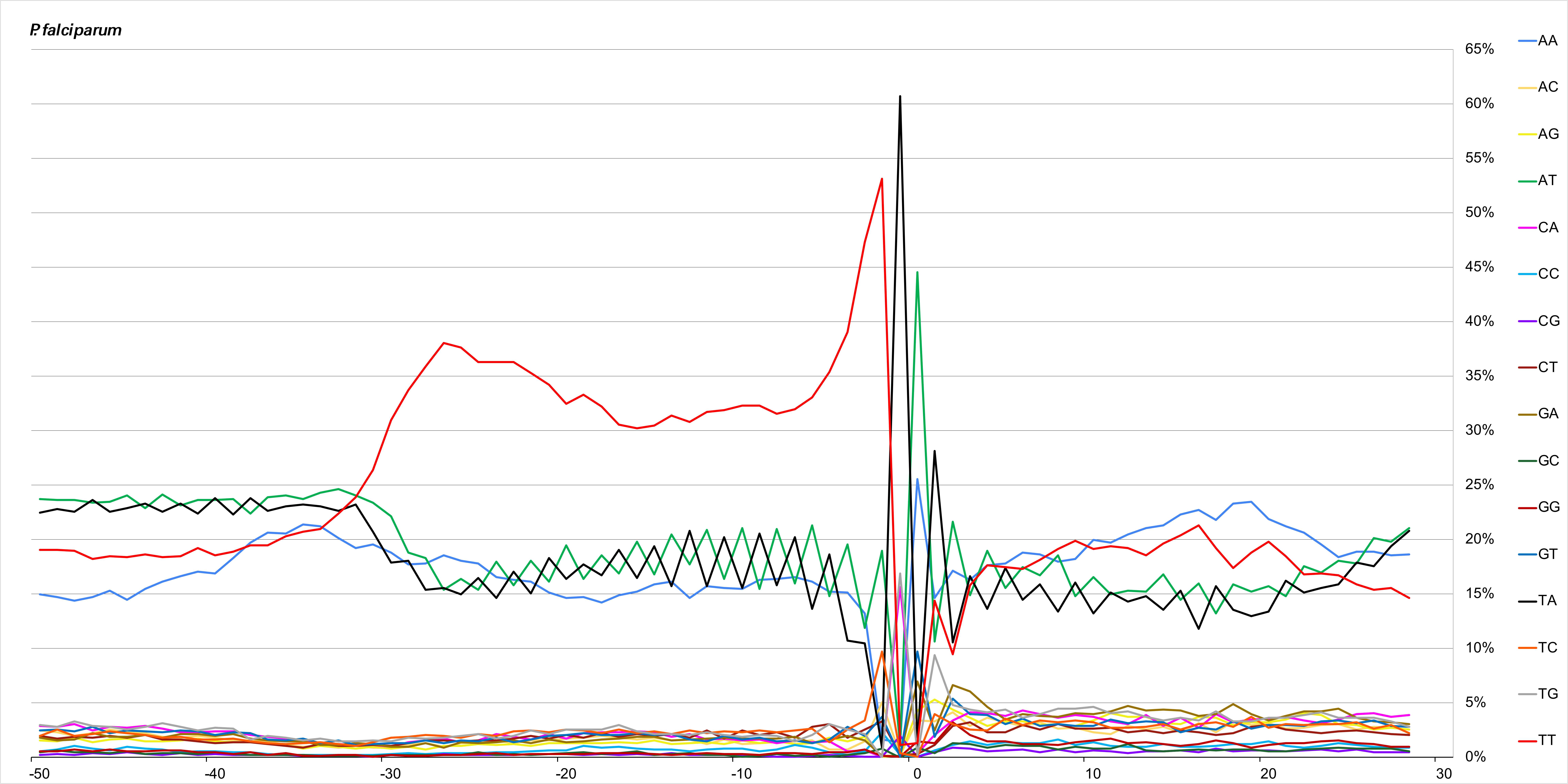
P. falciparum
65%
60%
55%
50%
45%
40%
35%
30%
25%
20%
15%
10%
5%
0%
-50
-40
-30
-20
-10
0
10
20
30
AA
AC
AG
AT
CA
CC
CG
CT
GA
GC
GG
GT
TA
TC
TG
TT
