# Supplementary material for: Evolutionary Invariant of the Structure of DNA Double Helix in RNAP II Core Promoters
Source: Int J Mol Sci. 2022 Sep 17;23(18):10873. doi: 10.3390/ijms231810873 (PMC9504043; doi:10.3390/ijms231810873)
Supplement: Supplementary file 1 [file ijms-23-10873-s001.zip › s3.pdf]

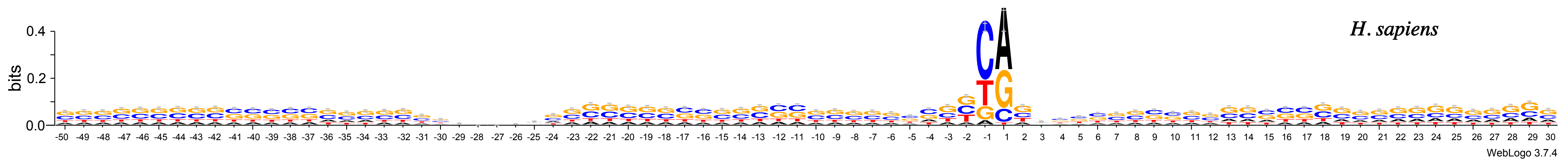
H. sapiens
bits
WebLogo 3.7.4

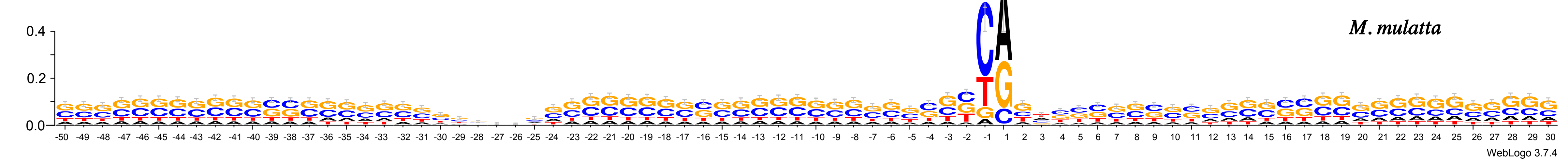

M. mulatta
0.4
0.2
0.0
WebLogo 3.7.4

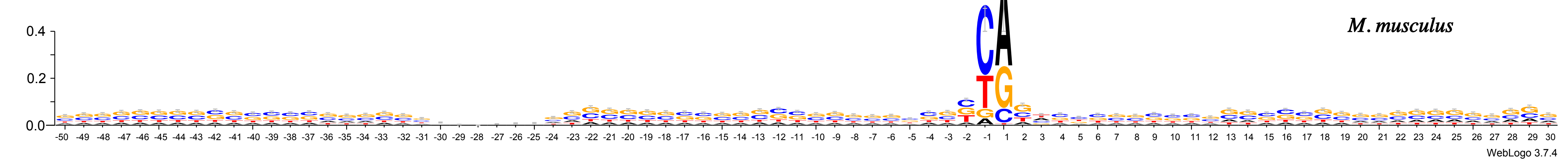
M. musculus
WebLogo 3.7.4

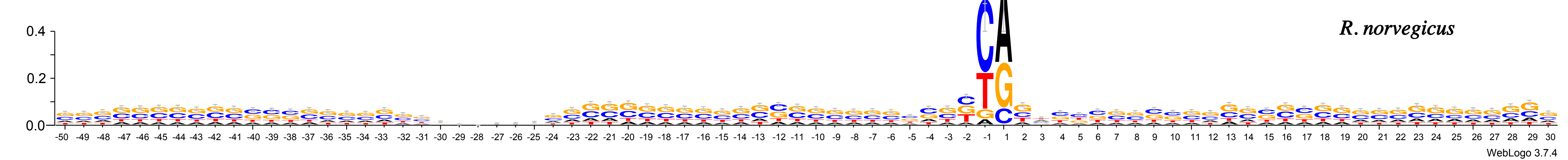
R. norvegicus
0.4
0.2
0.0
-50 -49 -48 -47 -46 -45 -44 -43 -42 -41 -40 -39 -38 -37 -36 -35 -34 -33 -32 -31 -30 -29 -28 -27 -26 -25 -24 -23 -22 -21 -20 -19 -18 -17 -16 -15 -14 -13 -12 -11 -10 -9 -8 -7 -6 -5 -4 -3 -2 -1 1 2 3 4 5 6 7 8 9 10 11 12 13 14 15 16 17 18 19 20 21 22 23 24 25 26 27 28 29 30
WebLogo 3.7.4

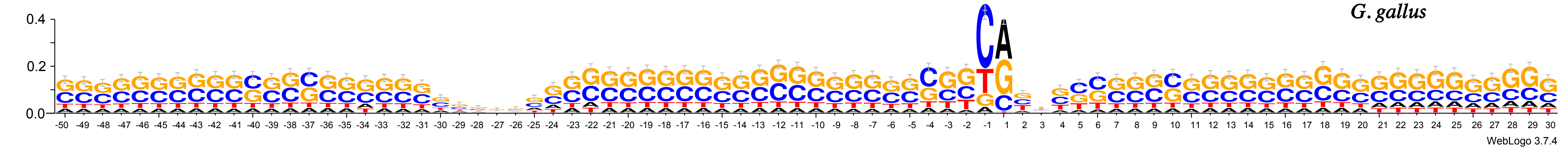
G. gallus
0.4
0.2
0.0
-50 -49 -48 -47 -46 -45 -44 -43 -42 -41 -40 -39 -38 -37 -36 -35 -34 -33 -32 -31 -30 -29 -28 -27 -26 -25 -24 -23 -22 -21 -20 -19 -18 -17 -16 -15 -14 -13 -12 -11 -10 -9 -8 -7 -6 -5 -4 -3 -2 -1 1 2 3 4 5 6 7 8 9 10 11 12 13 14 15 16 17 18 19 20 21 22 23 24 25 26 27 28 29 30
WebLogo 3.7.4

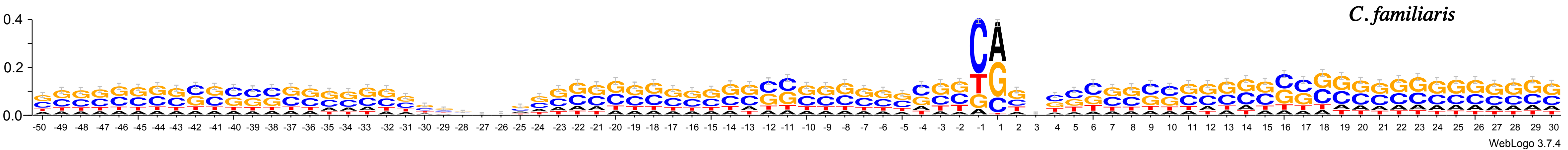
C. familiaris
0.4
0.2
0.0
-50 -49 -48 -47 -46 -45 -44 -43 -42 -41 -40 -39 -38 -37 -36 -35 -34 -33 -32 -31 -30 -29 -28 -27 -26 -25 -24 -23 -22 -21 -20 -19 -18 -17 -16 -15 -14 -13 -12 -11 -10 -9 -8 -7 -6 -5 -4 -3 -2 -1 1 2 3 4 5 6 7 8 9 10 11 12 13 14 15 16 17 18 19 20 21 22 23 24 25 26 27 28 29 30
WebLogo 3.7.4

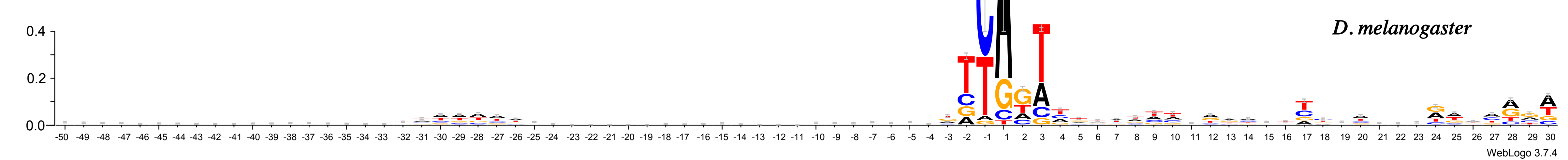
D. melanogaster
0.4
0.2
0.0
-50 -49 -48 -47 -46 -45 -44 -43 -42 -41 -40 -39 -38 -37 -36 -35 -34 -33 -32 -31 -30 -29 -28 -27 -26 -25 -24 -23 -22 -21 -20 -19 -18 -17 -16 -15 -14 -13 -12 -11 -10 -9 -8 -7 -6 -5 -4 -3 -2 -1 1 2 3 4 5 6 7 8 9 10 11 12 13 14 15 16 17 18 19 20 21 22 23 24 25 26 27 28 29 30
WebLogo 3.7.4

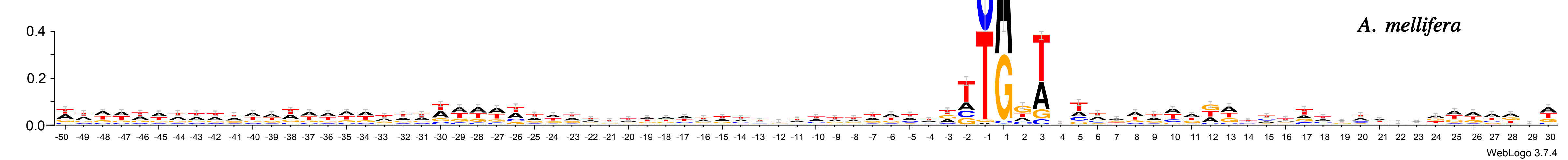
A. mellifera
0.4
0.2
0.0
-50 -49 -48 -47 -46 -45 -44 -43 -42 -41 -40 -39 -38 -37 -36 -35 -34 -33 -32 -31 -30 -29 -28 -27 -26 -25 -24 -23 -22 -21 -20 -19 -18 -17 -16 -15 -14 -13 -12 -11 -10 -9 -8 -7 -6 -5 -4 -3 -2 -1 1 2 3 4 5 6 7 8 9 10 11 12 13 14 15 16 17 18 19 20 21 22 23 24 25 26 27 28 29 30
WebLogo 3.7.4

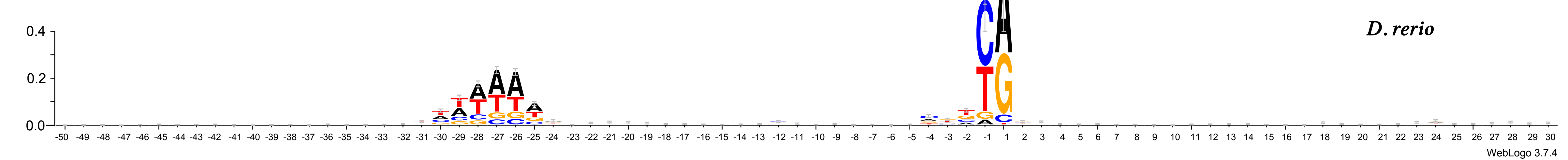
D. rerio
0.4
0.2
0.0
-50 -49 -48 -47 -46 -45 -44 -43 -42 -41 -40 -39 -38 -37 -36 -35 -34 -33 -32 -31 -30 -29 -28 -27 -26 -25 -24 -23 -22 -21 -20 -19 -18 -17 -16 -15 -14 -13 -12 -11 -10 -9 -8 -7 -6 -5 -4 -3 -2 -1 1 2 3 4 5 6 7 8 9 10 11 12 13 14 15 16 17 18 19 20 21 22 23 24 25 26 27 28 29 30
WebLogo 3.7.4

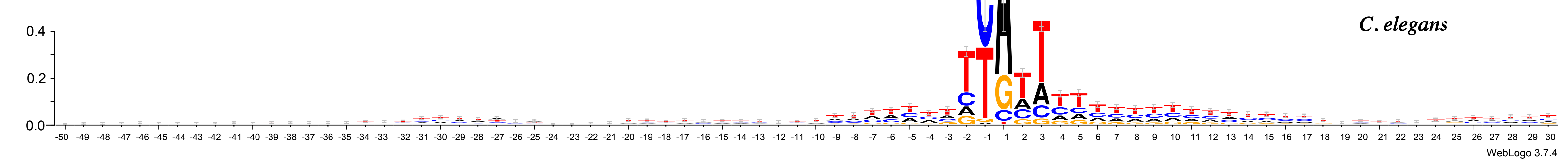

C. elegans
0.4
0.2
0.0
-50 -49 -48 -47 -46 -45 -44 -43 -42 -41 -40 -39 -38 -37 -36 -35 -34 -33 -32 -31 -30 -29 -28 -27 -26 -25 -24 -23 -22 -21 -20 -19 -18 -17 -16 -15 -14 -13 -12 -11 -10 -9 -8 -7 -6 -5 -4 -3 -2 -1 1 2 3 4 5 6 7 8 9 10 11 12 13 14 15 16 17 18 19 20 21 22 23 24 25 26 27 28 29 30
WebLogo 3.7.4

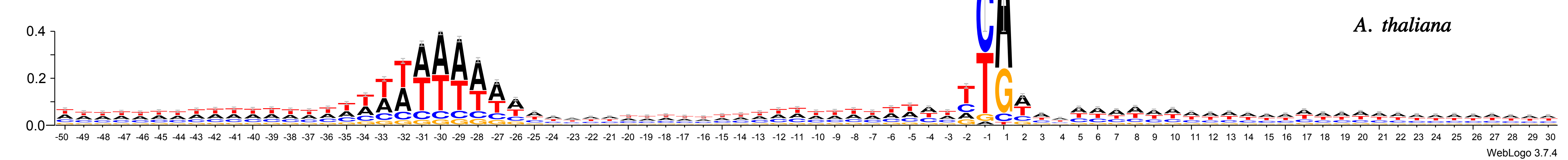
A. thaliana
0.4
0.2
0.0
-50 -49 -48 -47 -46 -45 -44 -43 -42 -41 -40 -39 -38 -37 -36 -35 -34 -33 -32 -31 -30 -29 -28 -27 -26 -25 -24 -23 -22 -21 -20 -19 -18 -17 -16 -15 -14 -13 -12 -11 -10 -9 -8 -7 -6 -5 -4 -3 -2 -1 1 2 3 4 5 6 7 8 9 10 11 12 13 14 15 16 17 18 19 20 21 22 23 24 25 26 27 28 29 30
WebLogo 3.7.4

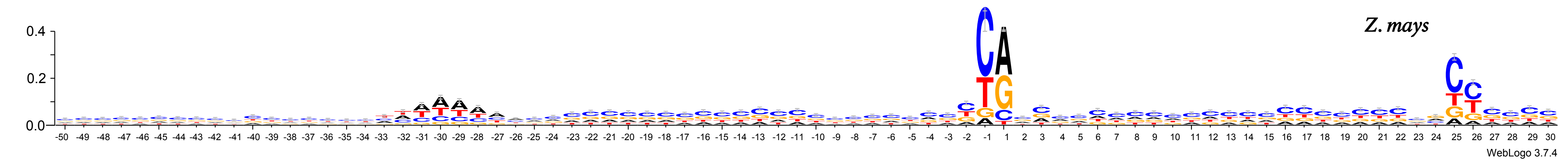

Z. mays
0.4
0.2
0.0
WebLogo 3.7.4

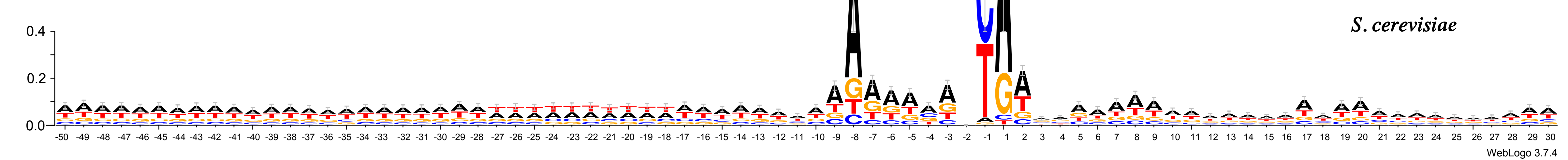
S. cerevisiae
0.4
0.2
0.0
-50 -49 -48 -47 -46 -45 -44 -43 -42 -41 -40 -39 -38 -37 -36 -35 -34 -33 -32 -31 -30 -29 -28 -27 -26 -25 -24 -23 -22 -21 -20 -19 -18 -17 -16 -15 -14 -13 -12 -11 -10 -9 -8 -7 -6 -5 -4 -3 -2 -1 1 2 3 4 5 6 7 8 9 10 11 12 13 14 15 16 17 18 19 20 21 22 23 24 25 26 27 28 29 30
WebLogo 3.7.4

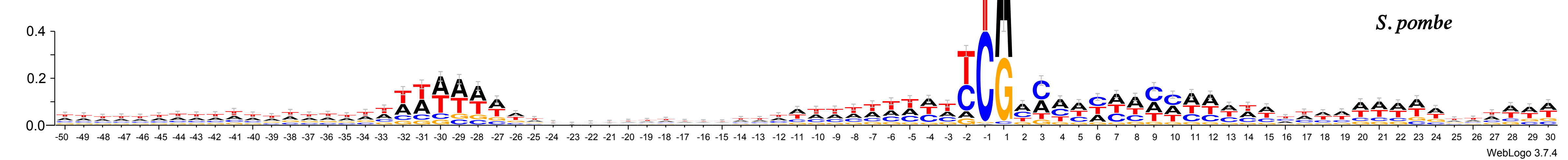
S. pombe
0.4
0.2
0.0
-50 -49 -48 -47 -46 -45 -44 -43 -42 -41 -40 -39 -38 -37 -36 -35 -34 -33 -32 -31 -30 -29 -28 -27 -26 -25 -24 -23 -22 -21 -20 -19 -18 -17 -16 -15 -14 -13 -12 -11 -10 -9 -8 -7 -6 -5 -4 -3 -2 -1 1 2 3 4 5 6 7 8 9 10 11 12 13 14 15 16 17 18 19 20 21 22 23 24 25 26 27 28 29 30
WebLogo 3.7.4

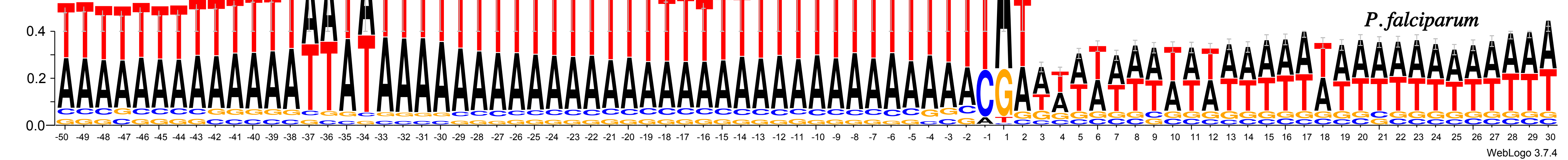
P.falciparum
WebLogo 3.7.4
