# Supplementary figures and images for: Evolutionary Invariant of the Structure of DNA Double Helix in RNAP II Core Promoters
Source: Int J Mol Sci. 2022 Sep 17;23(18):10873. doi: 10.3390/ijms231810873 (PMC9504043; doi:10.3390/ijms231810873)

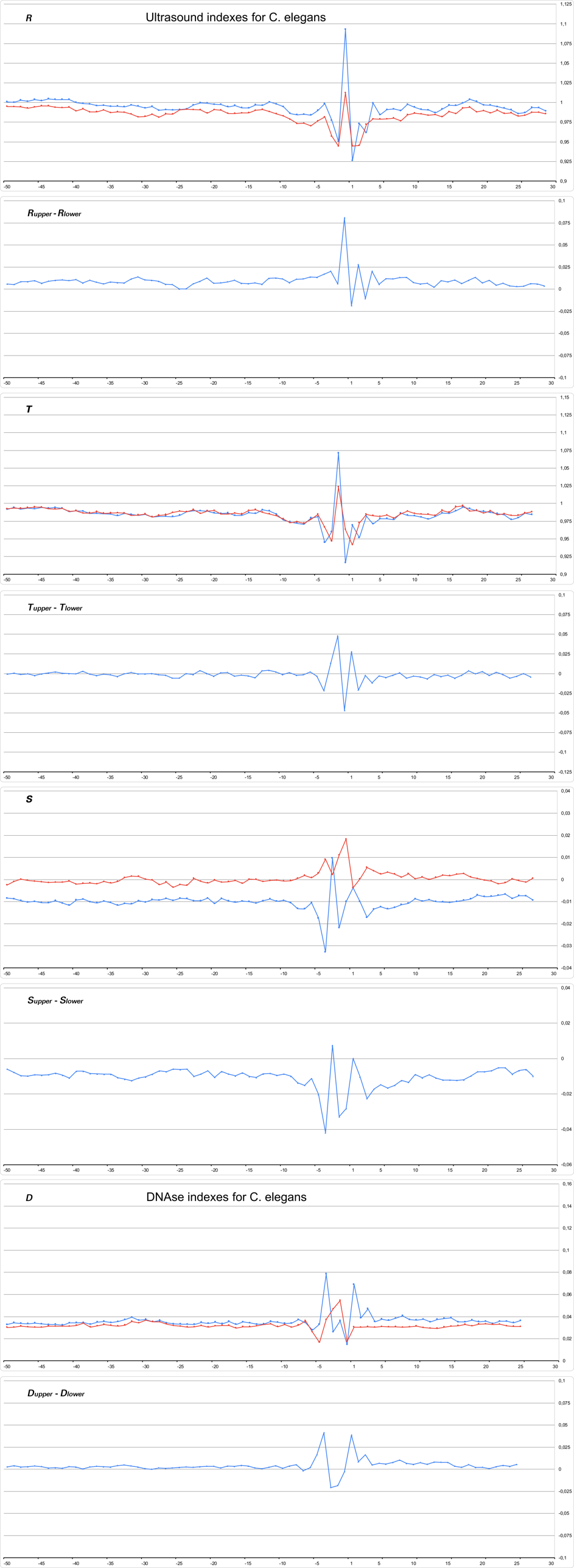

Supplement: Supplementary file 1 [file ijms-23-10873-s001.zip › s10.pdf]

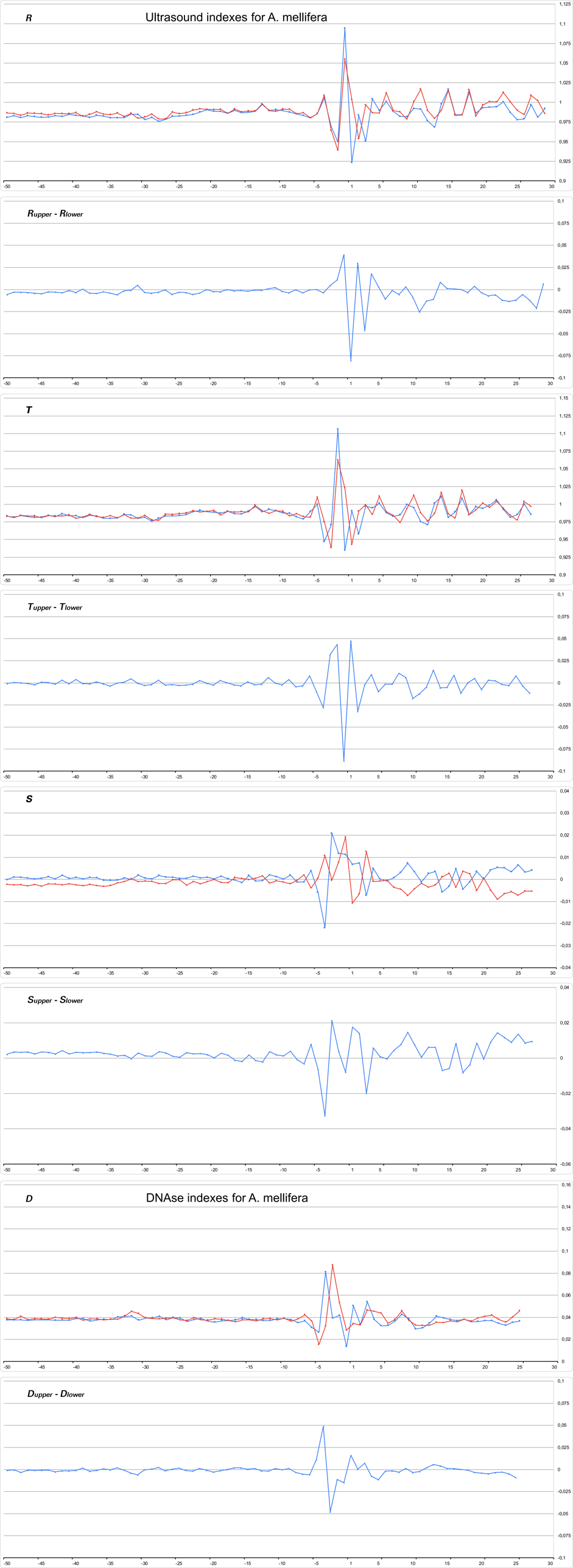

Supplement: Supplementary file 1 [file ijms-23-10873-s001.zip › s11.pdf]

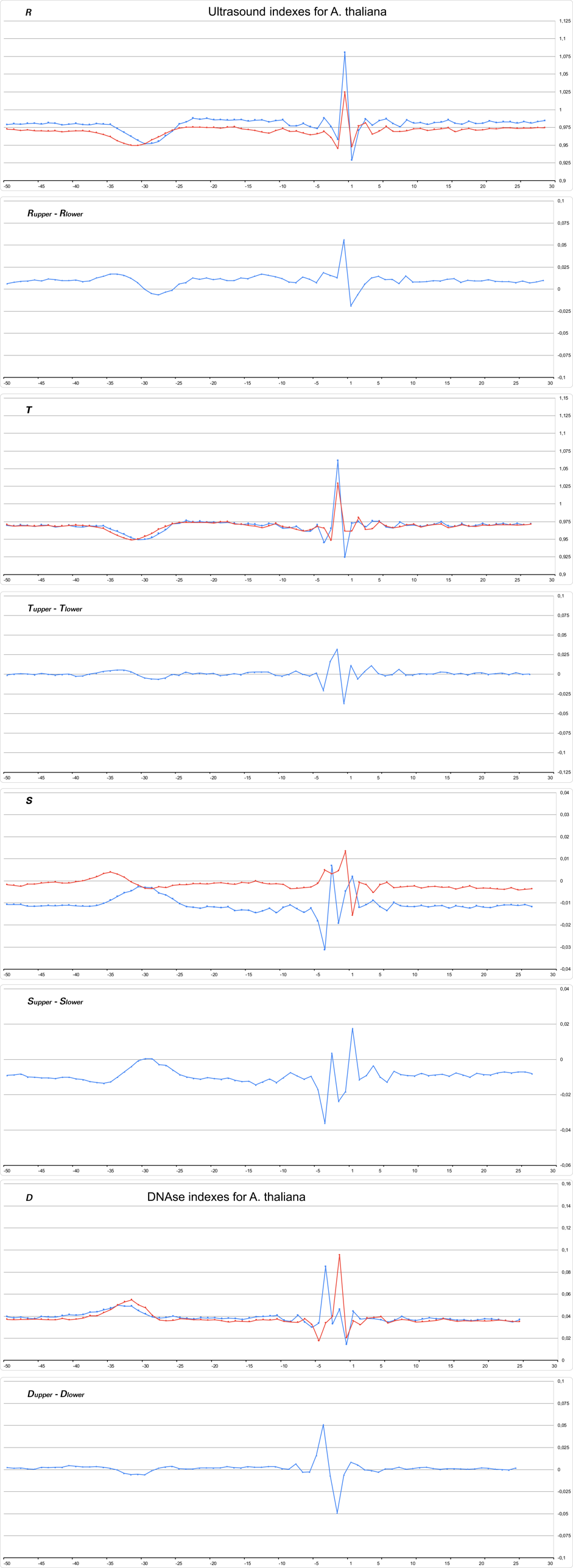

Supplement: Supplementary file 1 [file ijms-23-10873-s001.zip › s12.pdf]

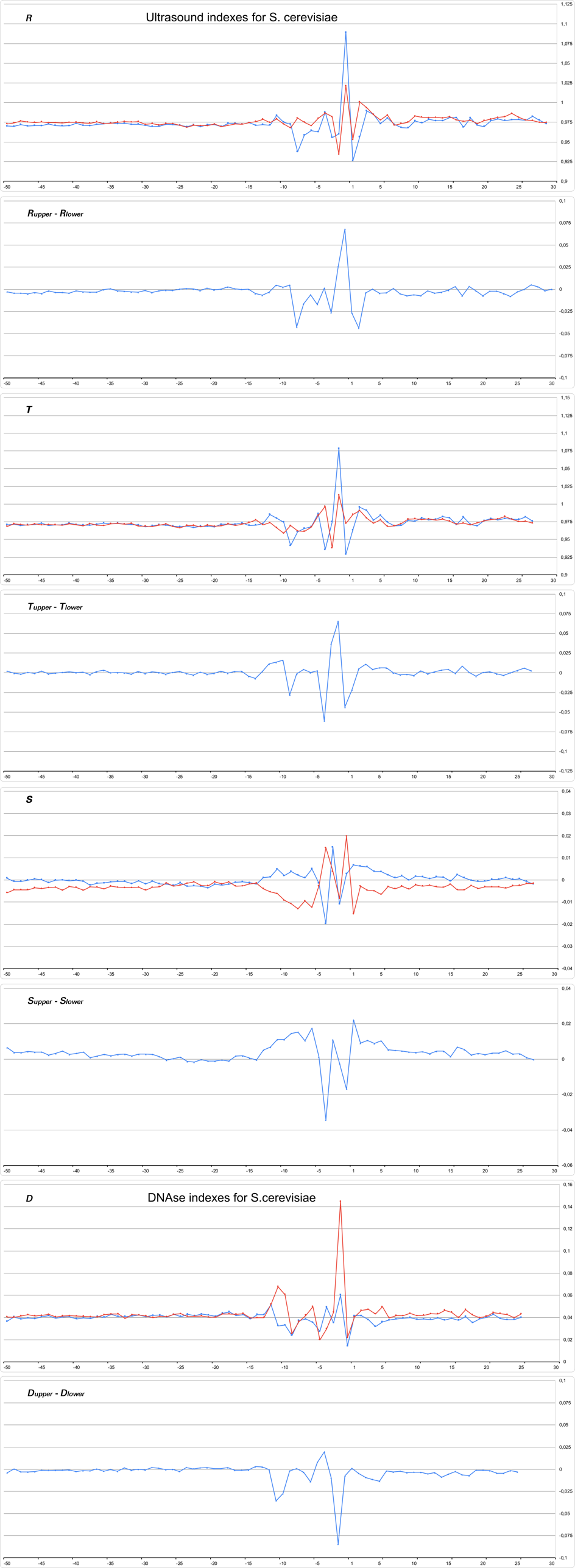

Supplement: Supplementary file 1 [file ijms-23-10873-s001.zip › s13.pdf]

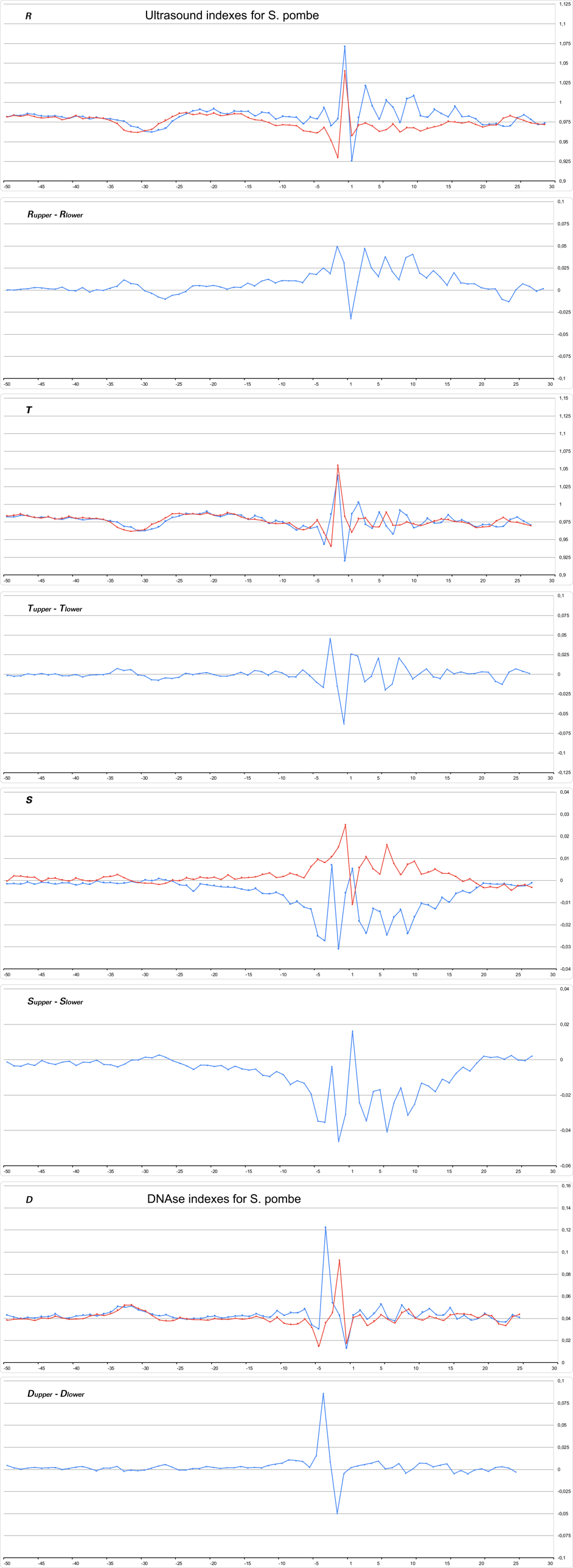

Supplement: Supplementary file 1 [file ijms-23-10873-s001.zip › s14.pdf]

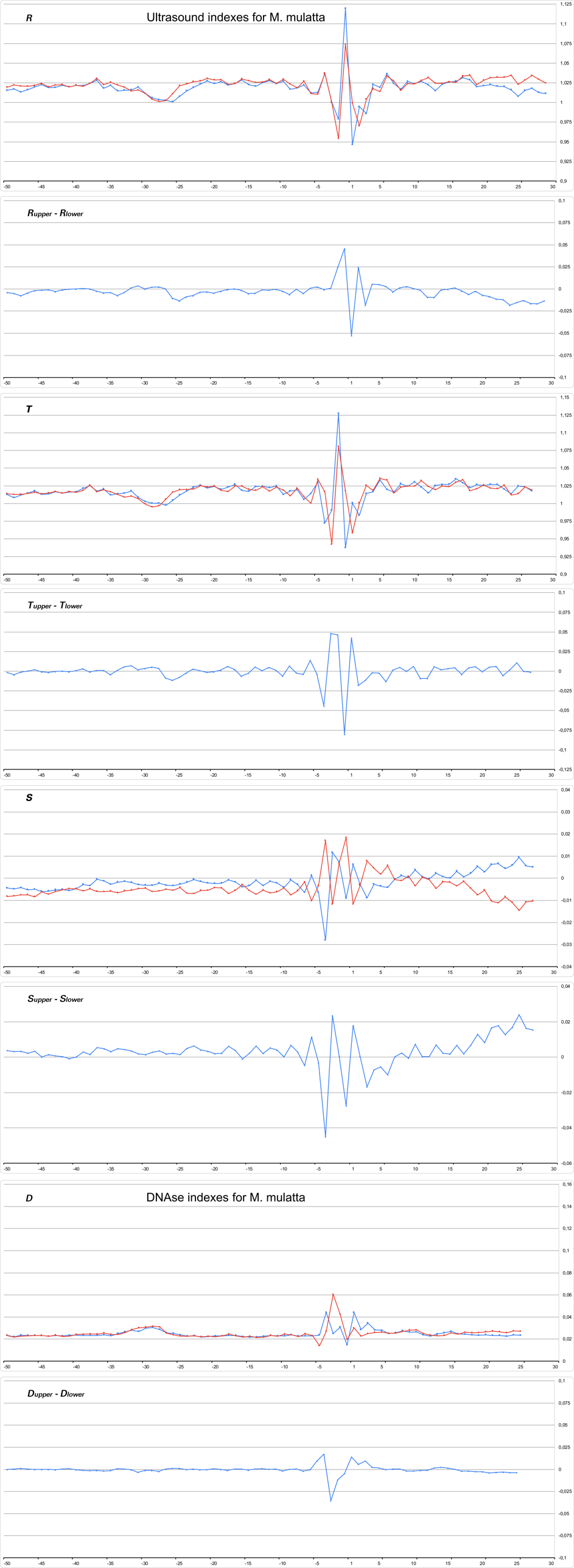

Supplement: Supplementary file 1 [file ijms-23-10873-s001.zip › s4.pdf]

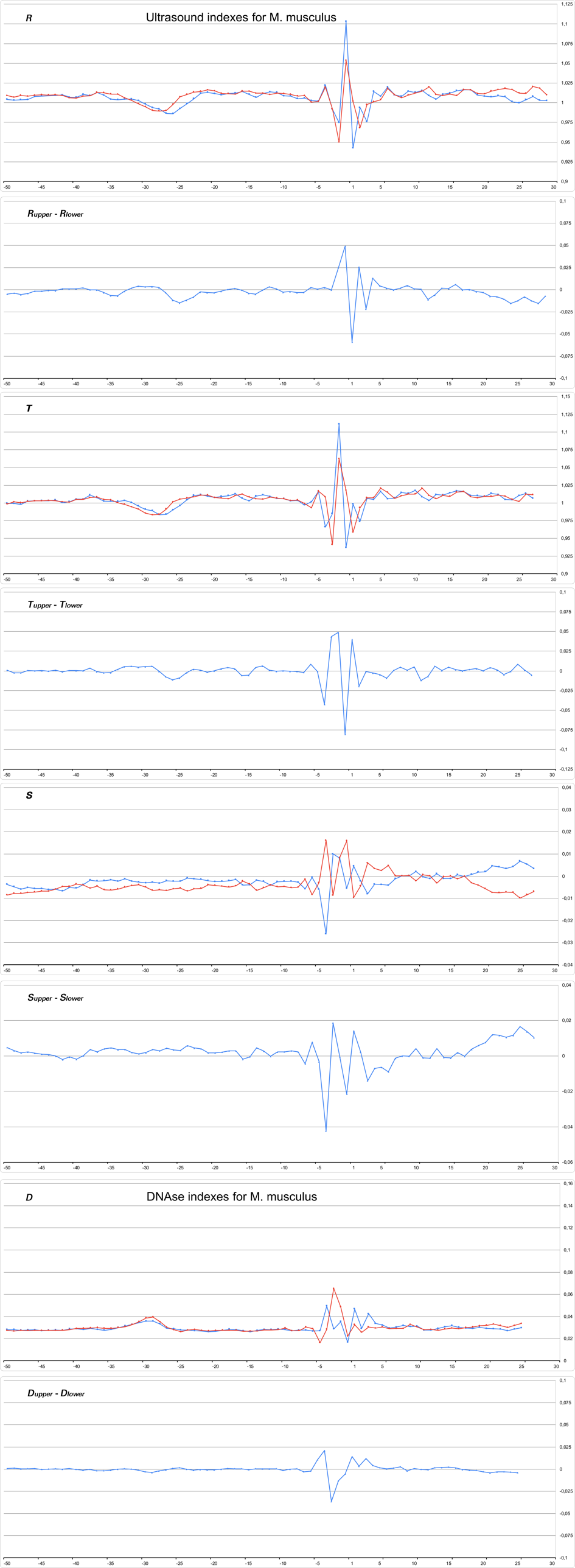

Supplement: Supplementary file 1 [file ijms-23-10873-s001.zip › s5.pdf]

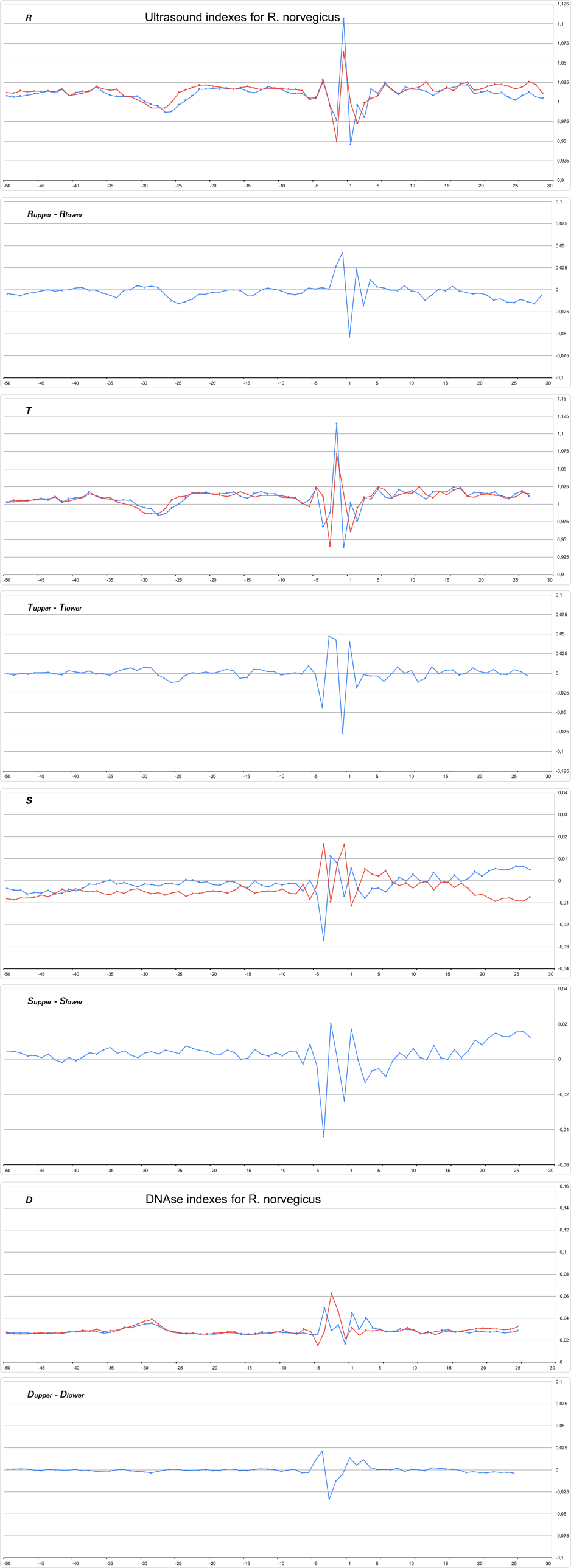

Supplement: Supplementary file 1 [file ijms-23-10873-s001.zip › s6.pdf]

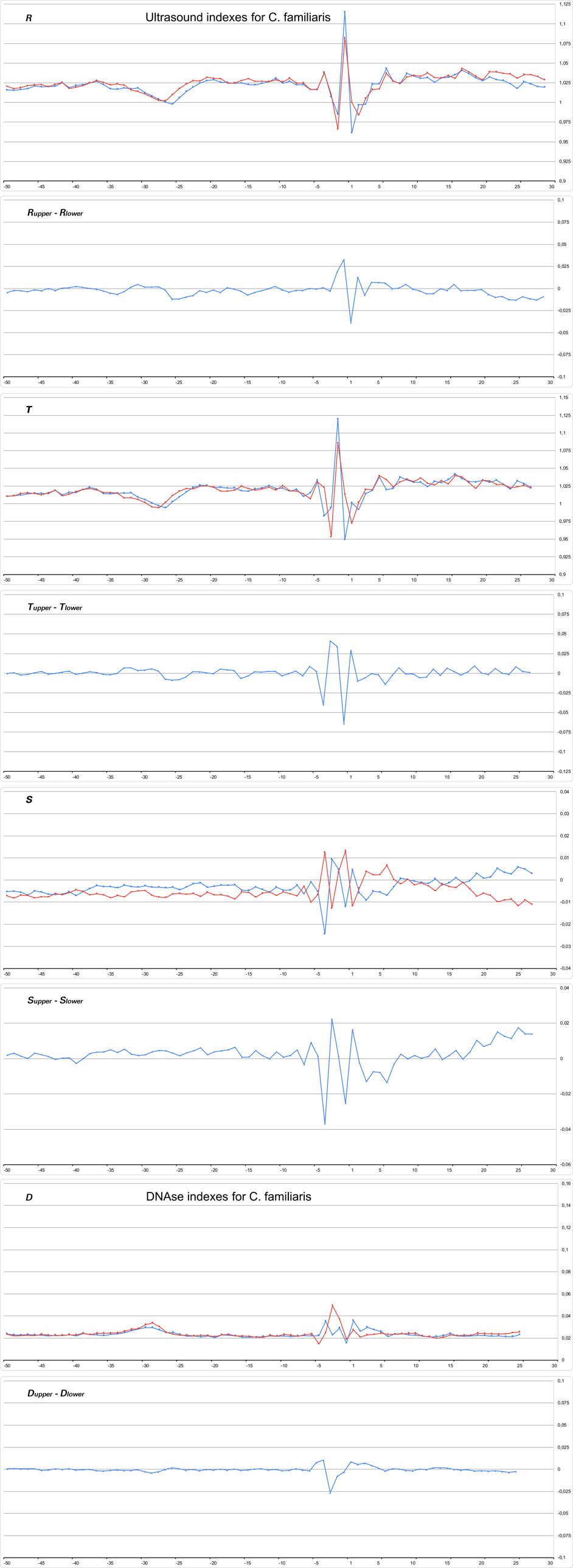

Supplement: Supplementary file 1 [file ijms-23-10873-s001.zip › s7.pdf]

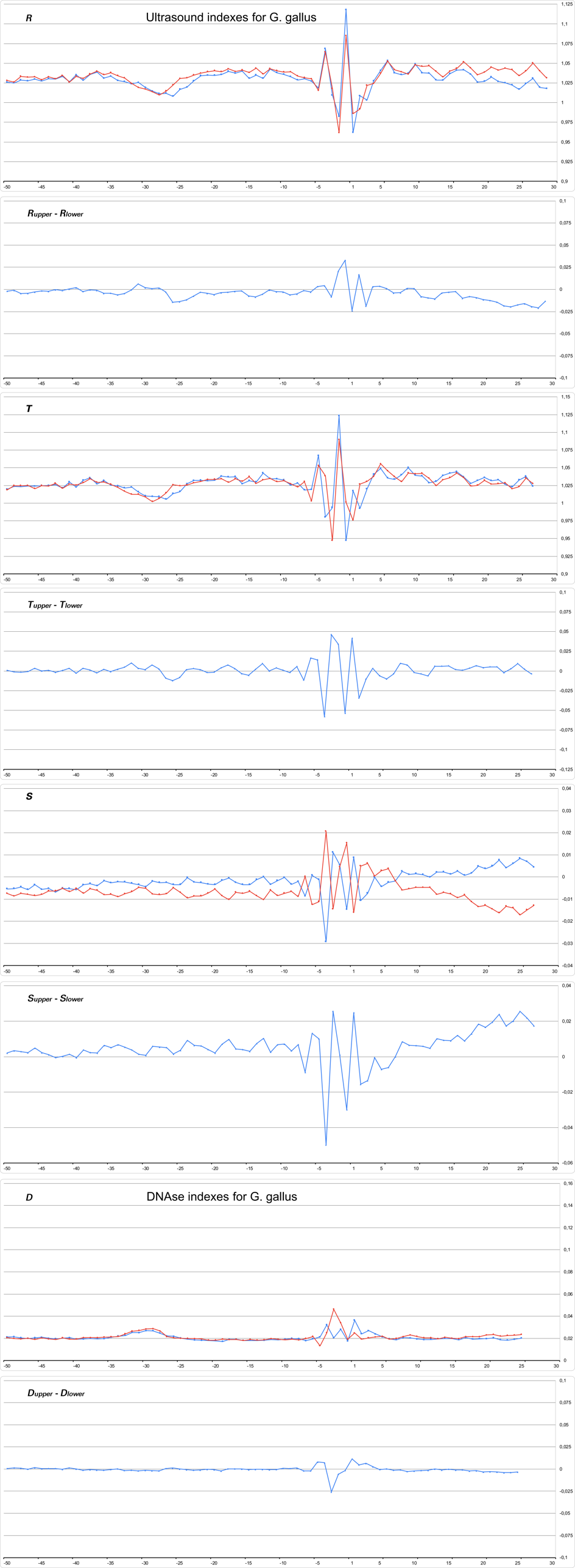

Supplement: Supplementary file 1 [file ijms-23-10873-s001.zip › s8.pdf]

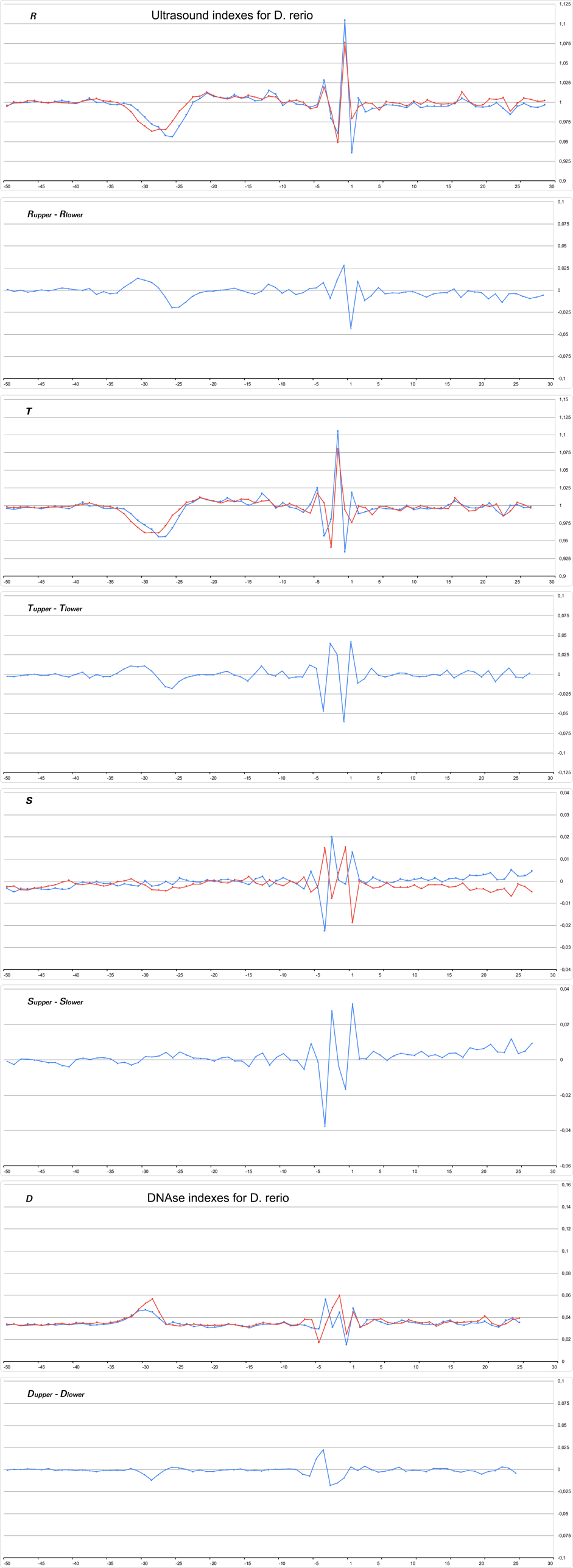

Supplement: Supplementary file 1 [file ijms-23-10873-s001.zip › s9.pdf]
