# Supplementary material for: Evolutionary Invariant of the Structure of DNA Double Helix in RNAP II Core Promoters
Source: Int J Mol Sci. 2022 Sep 17;23(18):10873. doi: 10.3390/ijms231810873 (PMC9504043; doi:10.3390/ijms231810873)
Supplement: Supplementary file 1 [file s2.pdf › s2.pdf]

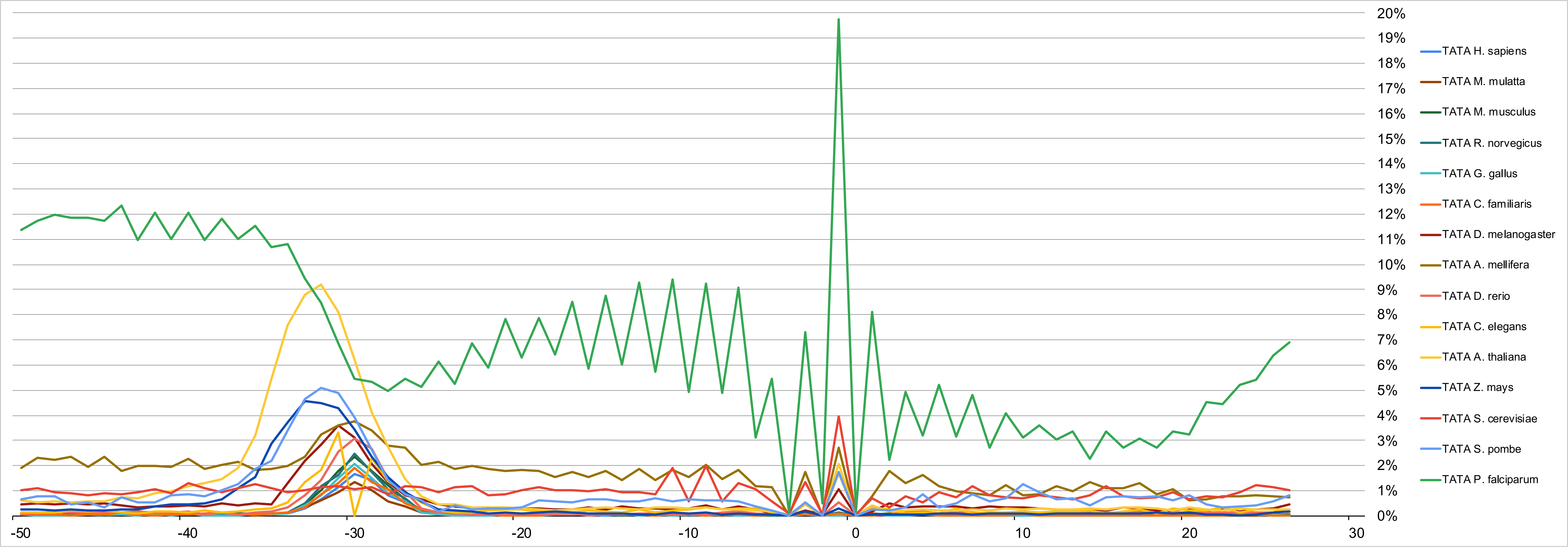
20%
19%
18%
17%
16%
15%
14%
13%
12%
11%
10%
9%
8%
7%
6%
5%
4%
3%
2%
1%
0%
-50
-40
-30
-20
-10
0
10
20
30
TATA H. sapiens
TATA M. mulatta
TATA M. musculus
TATA R. norvegicus
TATA G. gallus
TATA C. familiaris
TATA D. melanogaster
TATA A. mellifera
TATA D. rerio
TATA C. elegans
TATA A. thaliana
TATA Z. mays
TATA S. cerevisiae
TATA S. pombe
TATA P. falciparum

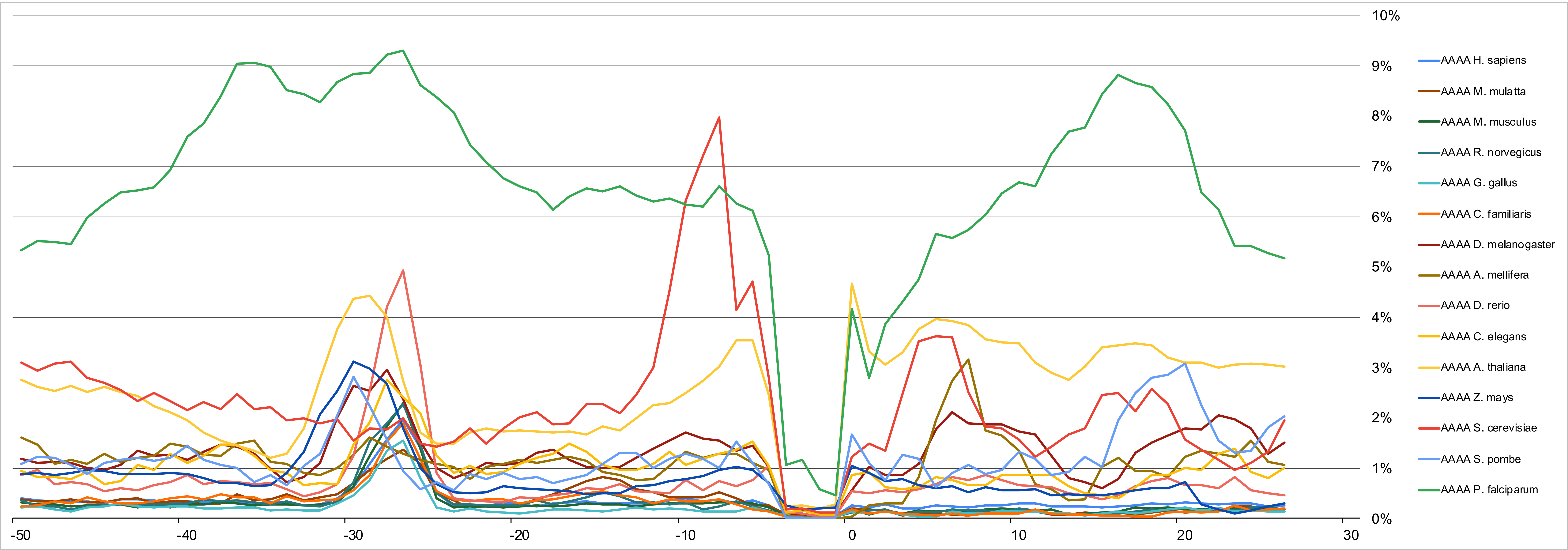
10%
9%
8%
7%
6%
5%
4%
3%
2%
1%
0%
-50
-40
-30
-20
-10
0
10
20
30
AAAA H. sapiens
AAAA M. mulatta
AAAA M. musculus
AAAA R. norvegicus
AAAA G. gallus
AAAA C. familiaris
AAAA D. melanogaster
AAAA A. mellifera
AAAA D. rerio
AAAA C. elegans
AAAA A. thaliana
AAAA Z. mays
AAAA S. cerevisiae
AAAA S. pombe
AAAA P. falciparum
